# Mixed pyruvate labeling enables backbone resonance assignment of large proteins using a single experiment

**DOI:** 10.1038/s41467-017-02767-8

**Published:** 2018-01-24

**Authors:** Scott A. Robson, Koh Takeuchi, Andras Boeszoermenyi, Paul W. Coote, Abhinav Dubey, Sven Hyberts, Gerhard Wagner, Haribabu Arthanari

**Affiliations:** 1 Department of Biochemistry and Molecular Pharmacology, Harvard Medical School, Boston, MA 02115 USA; 2 Molecular Profiling Research Center for Drug Discovery, National Institute of Advanced Industrial Science and Technology, 2-3-26 Aomi, Koto-ku, Tokyo 135-0064 Japan; 3 Dana-Farber Cancer Institute, 450 Brookline Ave, Boston, MA 02215 USA

## Abstract

Backbone resonance assignment is a critical first step in the investigation of proteins by NMR. This is traditionally achieved with a standard set of experiments, most of which are not optimal for large proteins. Of these, HNCA is the most sensitive experiment that provides sequential correlations. However, this experiment suffers from chemical shift degeneracy problems during the assignment procedure. We present a strategy that increases the effective resolution of HNCA and enables near-complete resonance assignment using this single HNCA experiment. We utilize a combination of 2-^13^C and 3-^13^C pyruvate as the carbon source for isotope labeling, which suppresses the one bond (^1^J_αβ_) coupling providing enhanced resolution for the Cα resonance and amino acid-specific peak shapes that arise from the residual coupling. Using this approach, we can obtain near-complete (>85%) backbone resonance assignment of a 42 kDa protein using a single HNCA experiment.

## Abstract (web summary)

Structure determination of large proteins by solution state NMR is challenging due to spectral overlap. Here the authors present a labeling strategy using 2-^13^C and 3-^13^C pyruvate as carbon source for *E. coli*, which increases the effective resolution of triple-resonance HNCA experiments and helps to overcome this problem.

## Introduction

Backbone resonance assignment is a necessary initial process in structural, dynamic and interaction studies of proteins by NMR. This has traditionally been achieved with a standard set of triple-resonance experiments^1^, supplemented by newer methods including hNcocaNH^2,3^, HncocaNH^4^ and the hNCAnH^5^. For small or homo-multimeric proteins, projection experiments such as APSY^6^ help resolve ambiguities in the chemical shift space by delineating a backbone spin system into high-dimensional correlations. However, most of these experiments harbor long-coherence transfer steps and are not optimal for large proteins (>40 kDa) due to relaxation-based loss of coherences. Deuteration in combination with TROSY-based backbone triple-resonance experiments is the standard practice to combat fast relaxation in large proteins^7,8^. However, multidimensional experiments with long-coherence transfer steps and multiple chemical shift labeling for ^13^C nuclei still suffer from relaxation loss before the detection period^9^.

Among the triple-resonance experiments, the HNCA^10^ is the most sensitive experiment that connects spin systems from internal to sequential residues. In the HNCA experiment, including the TROSY variation, the ^13^Cα coherence is transverse only during the chemical shift-encoding period, thus minimizing relaxation losses. In comparison, the sensitivity of the HNCACB experiment is theoretically and experimentally approximately one-fourth to that of the HNCA experiment, for high molecular weight proteins at high field^9^. The HNCA has all the necessary and sufficient information to complete sequence specific assignment. In practice, however, unique assignment of all backbone spin systems from this experiment alone is not possible due to insufficient dispersion of the ^13^Cα chemical shifts, resulting in multiple degeneracies in peak positions. This degeneracy scales with the protein size and concomitant increase in the number of spins within the given spectral space. This degeneracy problem can be alleviated by increasing resolution in the ^13^Cα dimension. Since the dipole-dipole interaction between the ^13^Cα and its attached ^1^Hα is the main source of ^13^Cα relaxation, deuteration of a protein is the immediate way to improve resolution of ^13^Cα signals (Supplementary Figure 1)^11,12^. Theoretical calculations reveal that the transverse relaxation rate of ^13^Cα in the deuterated case is ~8 times slower than in the non-deuterated case. The estimated line widths of ^13^Cα that can be achieved for 40 and 80 kDa deuterated proteins at 800 MHz are 4.4 and 8.8 Hz, respectively, at 298 K (Supplementary Figure 1). Narrow ^13^Cα line widths can be obtained with a relatively long indirect evolution period (>2 times T_2_), which corresponds to a maximal ^13^Cα evolution time of 114 ms (684 complex points for 4.4 Hz digital resolution) and 57 ms (342 complex points for 8.8 Hz digital resolution) assuming a ^13^Cα sweep width of 6000 Hz and one zero fill prior to the Fourier transform. Experimentally, correlation times (τ_c_) of two large NMR model systems, Maltose Binding Protein^13^ (MBP, 42 kDa) and Malate Synthase G protein^14^ (MSG, 82 kDa), have been determined using ^15^N relaxation measurements. MBP and MSG have τ_c_ values of 16.2 ns and 36 ns, respectively, at 310 K. These correlation times give theoretical ^13^Cα line widths of 3.0 Hz and 6.6 Hz at a magnetic field of 800 MHz, assuming the proteins are deuterated. While these resolutions cannot be obtained in a reasonable time with uniform sampling of the Nyquist grid, they can be theoretically achieved using non-uniform sampling (NUS)^15–17^. Although deuteration coupled with NUS permits access to high-resolution (4–8 Hz) in the ^13^Cα dimension, in practice, this resolution cannot be achieved because of the inherent Cα–Cβ coupling (^1^J_αβ_), which limits the resolution to 35–40 Hz. This can be seen in the 2D plane of an HNCA experiment on the Protein G B1 domain (GB1)^18^, uniformly labeled using ^13^C-glucose (U-^13^C ^2^H glucose) and deuterated. (Supplementary Figure 2A).

There have been several efforts to combat this resolution limit including band selective ^13^Cβ decoupling^19^, post-acquisition virtual decoupling,^20^ and constant time evolution^21^. Each of these techniques have their own drawbacks. Selective ^13^Cβ decoupling often affects the Cα resonances of glycine, serine, threonine, and proline residues due to their proximity to the decoupled frequencies (Supplementary Figure 2B). Virtual decoupling demands a uniform ^1^J_αβ_ coupling across all spin systems and even small differences (~3 Hz) from the chosen deconvolution value will result in non-ideal line shapes, especially when long acquisition times are employed (Supplementary Figure 2C-F). While the constant time (CT) approach removes the coupling, it comes at a substantial cost of sensitivity due to the long delays (~60 ms (2xCT) or ~90 ms (3xCT)) required to achieve high resolution. In constant time experiments, the initial point of the free induction decay (FID) experiences relaxation losses corresponding to the entire CT period^22^. This loss in sensitivity can be seen even for small proteins like GB1 (Supplementary Figure 2G) and is prohibitive for large proteins.

Yet another approach to remove this ^1^J_αβ_ coupling is to use the Stereo-Array Isotope Labeling (SAIL) strategy^23^ or to use 1-^13^C-labeled or 2-^13^C-labeled glucose as the carbon source^24^. The former approach is expensive, the latter suffers from a low maximum labeling rate (<50%). Given these challenges, engineering an inexpensive and efficient way of labeling proteins with ^13^C at the Cα position and ^12^C at Cβ would vastly improve the resolution of the sensitive HNCA experiment.

**Fig. 1:**
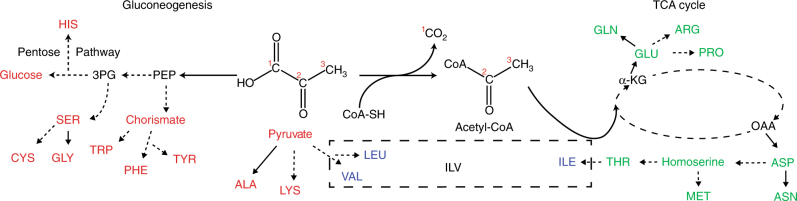
The principal metabolic pathways that lead from pyruvate to the amino acids. Direct conversion between molecules is indicated with solid lines while pathways involving one or more intermediates is indicated with dashed lines. With pyruvate as the only carbon source, gluconeogenesis (left) results in “directly” synthesized amino acids (red). The pyruvate carbons are numbered with red numerals. The TCA-derived amino acids (right, green) are synthesized when acetyl-CoA from pyruvate enters the cycle. The ILV amino acids (dashed box) derive their Cβ atom from more complicated metabolism as discussed in the main text

Isotopic labeling using amino acid precursors such as glycerol and pyruvate has been previously proposed to remove ^1^J_CC_ couplings^25–28^. When pyruvate is used as the only carbon source for *Escherichia coli* growth, it is metabolized via the gluconeogenesis pathway and the TCA cycle to produce amino acids (Fig. 1). Eleven amino acids (Fig. 1; red, LEU and VAL) are either synthesized directly from pyruvate or semi-directly via the gluconeogenesis pathway. These “direct” amino acids largely maintain the carbon backbone structure of pyruvate. Specifically, the C1, C2, and C3 carbons (Fig. 1) of pyruvate are incorporated into the CO, Cα, and Cβ positions respectively, in these amino acids. Therefore, the amino acids synthesized from “direct” pathways will be ^13^C labeled at the Cα position and ^12^C labeled at Cβ, if 2-^13^C labeled pyruvate is used as the carbon source. Leucine and valine are exceptions in the group of “direct” amino acids. In leucine, CO and Cβ are incorporated from the C2 position of pyruvate, whereas its Cα is incorporated from the C3 position of pyruvate. In valine, both Cα and Cβ are incorporated from the C2 position of pyruvate. Thus, these amino acids are treated separately, along with isoleucine as discussed below (Fig. 1; blue). It should be noted that lysine is synthesized via condensation of pyruvate and an aspartate derivative, β-aspartyl-4-semialdehyde. Since aspartate is an amino acid derived from the TCA cycle, the Cα and Cβ positions of lysine retain the labeling pattern of pyruvate. Thus, we treat lysine as a “direct” amino acid.

The remaining nine amino acids (Fig. 1, green) are synthesized from pyruvate via the TCA cycle. Asparagine, aspartate, isoleucine, methionine and threonine are synthesized from oxaloacetate (Fig. 1). Arginine, glutamine, glutamate and proline are from α-ketoglutarate (Fig. 1). For these amino acids, the C3 position of pyruvate would have a higher chance of being incorporated at the Cα position as compared to the C2 position. In addition, the TCA cycle scrambles the ^13^C-labeled positions for these TCA-derived amino acids, depending on the number of times the amino acid precursors have passed through the TCA cycle before incorporation into amino acids. Furthermore, the TCA cycle reintroduces the ^13^C–^13^C coupling by directly condensing oxaloacetate with acetyl-CoA at the equivalent C3 position from pyruvate. After three cycles through the TCA, a certain population of “TCA-derived” amino acids are doubly ^13^C labeled in both Cα and Cβ positions when 3-^13^C pyruvate is used.

Like other branched amino acids, isoleucine is an exception. Isoleucine is synthesized via condensation of a threonine derivative, 2-oxobutyric acid, and pyruvate at C2 position. This results in the Cβ position of isoleucine being exclusively incorporated from the C2 carbon of pyruvate. On the other hand, the ^13^Cα labeling rate would be the same as threonine. Therefore, Cα and Cβ of isoleucine would both be ^13^C labeled when 2-^13^C pyruvate is used as a carbon source.

We explored the use of deuterated ^13^C pyruvate as a carbon source with the intention of obtaining specific labeling patterns where uncoupled ^13^Cα nuclei could be generated, which will result in narrow lines to aid backbone assignment from a single HNCA experiment. We have acquired high-resolution HNCA spectra on pyruvate-labeled proteins up to 42 kDa using non-uniform sampling in 3–4 days. These spectra reveal very narrow, high-resolution lines in the Cα dimension, for all amino acids and can resolve systems well below the 35–40 Hz ^1^J_αβ_ carbon coupling. In addition, we show that the residual coupling, quantified by the “coupled to uncoupled peak height ratio” (C2UR), is an indicator of the metabolic origin of the amino acid and provides information about the matching sequential system. Finally, we demonstrate that the peak shape along the Cα dimension, along with the narrow, high-resolution lines, can be used to make extensive sequence specific assignments from the HNCA experiment alone.

## Results

### Incorporation of ^13^C labeling in amino acids from pyruvate

The theoretical ^13^C-labeling patterns with 2-^13^C pyruvate and 3-^13^C pyruvate were experimentally verified by labeling proteins in growth media with D_2_O as the solvent, ammonium chloride as the ^15^N source and pyruvate as the ^13^C source (see Experimental section). The difference in the labeling pattern between “direct” and “TCA derived” amino acids is exemplified by the A22 and E21 resonances, respectively, of the protein GB1 in a high-resolution HNCA experiment with ^13^CO decoupling (Supplementary Figure 3). As expected, labeling with 2-^13^C pyruvate strongly incorporates ^13^C at the Cα position of alanine, an amino acid derived directly from pyruvate. There is no ^1^J_αβ_ coupling present indicating that the Cβ is not ^13^C labeled. On the other hand, glutamate is poorly labeled at the Cα position by 2-^13^C pyruvate, and there is no apparent labeling at the Cβ position. When 3-^13^C pyruvate is used as the carbon source, glutamate is strongly labeled and alanine is poorly labeled at the Cα position. Interestingly, the height of coupled peaks of glutamate is just as intense as the central uncoupled peak. This indicates that glutamate is synthesized predominantly in a doubly, ^13^Cα and ^13^Cβ labeled, form after at least three cycles through the TCA. The ratio of “doubly ^13^C” (Cα and Cβ) labeled against singly ^13^Cα labeled glutamate is about 2:1. It should be noted that the ratio would vary depending on the expression condition and induction period, which influences the number of passes of the precursor through the TCA cycle. Hence a protein derived from individual expression conditions would have unique peak shape for its TCA-derived amino acids, which is dependent upon the expression conditions. The small but significant ^13^Cα labeling of alanine with 3-^13^C pyruvate is rather unexpected, but can be explained by a minor amino acid synthetic pathway via glycine that is produced via a condensation of CO_2_ and the methylene moiety of 5,10-methylene-THF that originates from the C3 position of pyruvate.

In summary, by using 2-^13^C pyruvate, the strong ^1^J_αβ_ coupling can be avoided for most of the “direct” amino acids, which provide access to sensitive and high-resolution resonances without splitting from the ^1^J_αβ_ carbon coupling. However, the ^13^C-labeling rate at Cα is low for “TCA-derived” amino acids, which, depending on the primary amino acid sequence would result in significant gaps in the backbone assignment efforts depending on the primary amino acid sequence. In contrast, 3-^13^C pyruvate would mainly label the Cα position of the “TCA-derived” amino acid and thus fill the gap left by using 2-^13^C pyruvate, however a substantial population would be doubly ^13^C labeled at the Cα and Cβ positions. Thus, in addition to the central uncoupled frequency, which can provide high-resolution matching of the spin systems, pyruvate labeling results in the emergence of the residual ^1^J_αβ_ coupling, which provides unique line shapes that are specific for each spin system, in exchange for some sensitivity.

### Generating resolution and amino acid-specific peak shapes

To concomitantly achieve sensitivity and the high-resolution enabled by 2-^13^C pyruvate with the unique labeling pattern and amino acid-specific line shape provided by 3-^13^C pyruvate, we decided to simultaneously use both 2-^13^C and 3-^13^C pyruvate. Along this line of thought, we tested two different approaches, post-mix and pre-mix. In the post-mix strategy, the protein was individually expressed with either 2-^13^C or 3-^13^C pyruvate as the carbon source, purified, and mixed in a 1:1 ratio to make the NMR sample. In contrast, in the pre-mix strategy we grew *E. coli* cultures by using an equal amount of 2-^13^C and 3-^13^C pyruvate as the carbon sources for protein expression. A graphical illustration of two strategies is shown in Supplementary Figure 3.

**Fig. 2:**
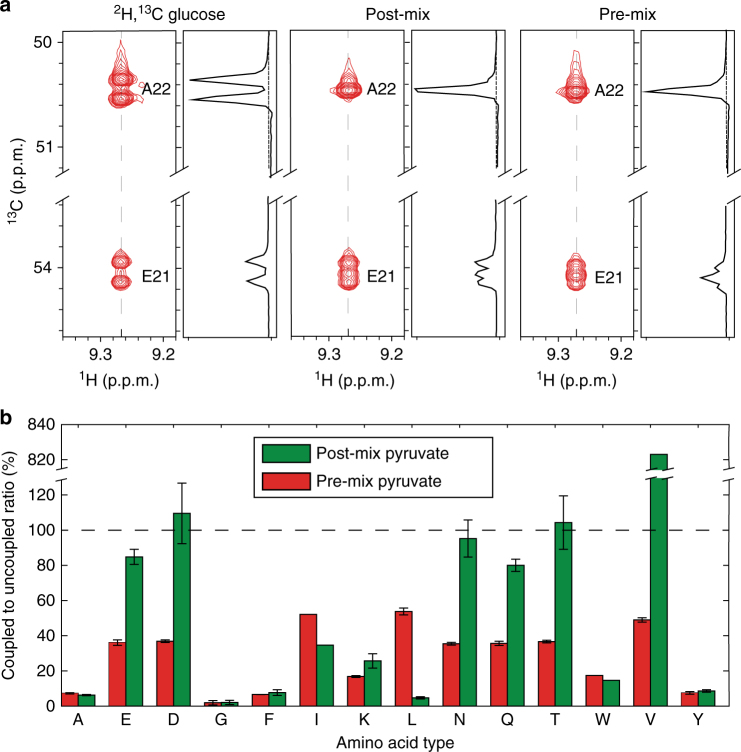
Comparison of post-mix and pre-mix 2-^13^C and 3-^13^C pyruvate samples. **a** HNCA strips for spin system A22 of GB1. [U-^2^H^13^C] glucose labeling (left) is compared to post-mix (middle) and pre-mix samples (right). **b** Comparison of coupled to uncoupled peak height ratio (C2UR) for post-mix (green) and pre-mix (red). The dashed line indicates a height of 100%, when the heights of the coupled to uncoupled peaks are equal. Error bars indicate the standard deviation of the C2UR ratio for a given amino acid, based on all occurrences of the amino acid in the primary sequence. Absence of error bars indicates only one occurrence of that amino acid in the sequence of GB1

In the post-mix strategy, the labeling pattern would be the simple arithmetic average of the individual 2-^13^C pyruvate and 3-^13^C pyruvate labeling. This sample maintains a strong uncoupled central peak for “direct” amino acids (A22), however the signal of the “TCA-derived” amino acid (E21) is still composed of equal height coupled and uncoupled peaks (Fig. 2a, post-mix). Although the coupling gives additional line shape information, the substantial population of coupled systems significantly reduces the sensitivity of the “TCA-derived” amino acids, as described above.

For the pre-mix strategy, the scrambling caused by the TCA cycle gives a distinct pattern, different from the post-mix sample. The labeling maintains a strong uncoupled central peak for “direct” amino acids (A22), while the signal of TCA-derived amino acid (E21) has a stronger uncoupled peak when compared to post-mix (Fig. 2a, pre-mix). This is because the oxaloacetate, which is derived from 3-^13^C pyruvate, would have a 50% chance of being condensed with the 2-^13^C labeled pyruvate-derived acetyl-CoA, which prevents the recoupling of the Cα and Cβ positions and dilutes the population of doubly labeled “TCA-derived” amino acids. These effects are clearly seen in the HNCA experiments utilizing pre-mix samples (Fig. 2a, pre-mix), in which pre-mix present a stronger, uncoupled peak when compared to post-mix (Fig. 2a, post-mix).

A fitting algorithm (Supplementary Figure 4) was used to estimate the coupled to uncoupled peak height ratio (C2UR) for all spin systems in GB1 for both post-mix and pre-mix GB1 samples (Fig. 2b). The post-mix strategy is reasonably good for some of the amino acids, especially the “direct” amino acids from the gluconeogenesis pathway. However, in other cases the post-mix condition is quite poor with ratios approaching 100%, indicating all three peaks are approximately the same height. In the worst case, valine, the intensity of the uncoupled peak is vanishingly small, which prevents meaningful extraction of the C2UR. In the pre-mix strategy, the central uncoupled peak is dominant when compare to the coupled peaks, for all amino acids types. Even in the worst cases of isoleucine, leucine, and valine, the coupled peaks are only half the height of the uncoupled peaks. This would allow us to use the central narrow uncoupled resonance for frequency matching. Thus, to take advantage of its more favorable labeling patterns, which result in high sensitivity and resolution, we decided to use the pre-mix strategy to acquire high-resolution HNCA experiments.

### Combining resolution and peak shape for backbone assignment

**Fig. 3:**
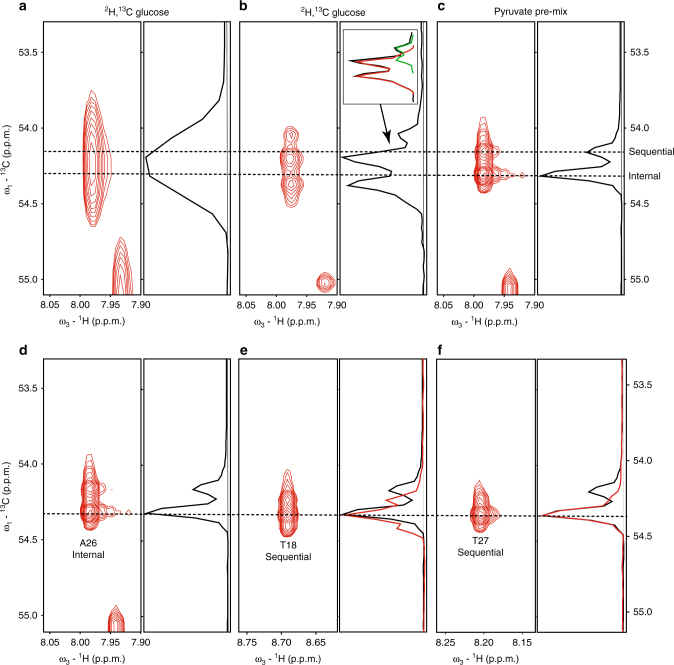
Enhanced resolution and peak shape with pre-mix samples solves overlap problems in GB1. All panels show a 2D plot of a spin system from a 3D HNCA spectrum (red contours) along with a 1D trace (black line) along the ^13^Cα dimension. **a** A spin system from uniformly ^13^C-labeled GB1 at standard HNCA resolution (~42 Hz). **b** Same protein sample as **a** with a higher resolution of ~5 Hz. The ^1^J_αβ_ coupling can be seen with overlap of the internal and sequential Cα peaks in this system (see 1D plot inset). **c** Same protein system but the sample is prepared using the 2-^13^C and 3-^13^C pyruvate pre-mix labeling strategy. The internal and sequential Cα peaks are now resolvable. **d** A repeat of **c** to clarify the comparison with **e** and **f**. The signal for the internal Cα of A26 is labeled. **e** An incorrect sequential system, T18, that matches perfectly for chemical shift but not for the peak shape. The 1D trace of sequential T18 signal (red) is overlaid with the A26 1D trace (black). **f** The correct sequential assignment, T27. The 1D sequential trace of T27 (red) perfectly matches the 1D internal trace of A26. 1D traces in **d**–**f** are plotted to equalize intensity to help with visual pattern matching

The utility and advantage of using pyruvate-labeled pre-mix samples and obtaining a high-resolution HNCA through Non-Uniform Sampling (NUS), in comparison to traditional HNCA spectra, becomes apparent even for a small protein like GB1. First, we would like to demonstrate the advantage of high resolution in removing degeneracies in Cα resonances. An HNCA experiment was acquired for the protein GB1 using NUS and the resulting spectrum was reconstructed with hmsIST^16^. 512 complex points were collected in the Cα dimension, spanning a sweep width of 6032 Hz, yielding a final digital resolution of ~5.8 Hz after zerofilling to 1024 points. In a separate processing effort, we truncated the ^13^Cα dimension of the above spectrum after reconstruction to obtain a digital resolution of ~42 Hz (corresponding to 72 complex points and zero filled to 128 points). This mimics the standard low-resolution practice that deliberately obscures the ^1^J_αβ_ coupling (Fig. 3a). The left panel shows a ^1^HN-^13^Cα strip for a system in GB1 with the right panel showing the 1D trace through the peak. Using all 512 complex points yields narrower peaks where the coupling can be seen (Fig. 3b). However, it is clear from the 2D strip and the 1D trace that this peak is composed of two sets of split peaks, one with higher intensity at low field, and the other with lower intensity at high field (see Fig. 3b, right panel, inset). No other resonances are present at or near the corresponding ^1^H/^15^N spin system in the HN plane of the spectrum, suggesting that this smaller peak is from the sequential Cα resonance and is overlapped with the intra-residue Cα resonance. Although the high resolution partially resolved the problem, the peaks cannot be readily identified if the coupling is present. In contrast, the same spectrum with identical parameters on a GB1 sample labeled with pre-mix 2-^13^C/3-^13^C pyruvate (Fig. 3c) shows two completely resolved uncoupled peaks, corresponding to the internal and sequential Cα resonances. Assignment through conventional means using multiple 3D experiments including HNCACB, have indicated that this system is A26/A25 and that these two peak shapes are consistent with the typical alanine peak shape we expect (Fig. 2). Thus, even for these two systems, which are about 34 Hz apart, one would easily resolve and identify the individual peaks by using the pre-mix strategy, but not with conventional uniform ^13^C labeling. This clearly demonstrates the utility of the ultra-high resolution achievable when the ^1^J_αβ_ coupling is removed.

The utility of the peak shape resulting from the residual ^1^J_αβ_ coupling is also demonstrated for the internal peak of A26 (Fig. 3d). Coincidentally, two systems provide a sequential match with their individual chemical shifts within the digital resolution of the spectrum (Fig. 3e, f). Therefore, even at this ultra-high resolution, we might not establish the unambiguous sequential connection from the chemical shift information alone. However, it is trivial to obtain the correct assignment by a mere inspection of the peak shape of the two sequential candidate systems. While the same line shape would be expected for the correct assignment, the first candidate fails to match the line shape of the internal A26 signal (Fig. 3e). On the other hand, the correct assignment shows a perfect line shape match between internal and sequential peaks (Fig. 3f).

To further test and validate the utility of labeling using pyruvate and the resolving power of pre-mix 2-^13^C and 3-^13^C pyruvate, we applied this strategy to assign the Maltose Binding Protein (MBP). MBP was labeled using the pre-mix strategy as outlined in the experimental methods. A high-resolution HNCA with TROSY selection in the ^1^H and ^15^N dimensions, was acquired on the sample with a final digital resolution of ~3.5 Hz for Cα. The sample concentration of the “pre-mix” MBP sample was 600 μM and the HNCA was recorded in ~4 days with 16 scans using NUS to sample ~9% of a 75(^15^N)×750(^13^C) grid (5216 total sampled points). This set up corresponded to an evolution time of 103 ms in the carbon dimension.

Every system present in the spectrum had a stronger central uncoupled peak (Supplementary Figure 5), which provided a precise peak position within a digital resolution of about 4.8 Hz, the expected line width for deuterated Cα in MBP at 310 K. The 2-^13^C and 3-^13^C pre-mix strategy gives rise to distinct peak shapes for each amino acid. This can be quantified by the C2UR, in the same way we compared the post-mix to pre-mix line shapes of amino acids above. The fitting algorithm extracts the C2UR for each amino acid type as the ratio *k*_2_/*k*_1_ where *k*_1_ and *k*_2_ is the height of the uncoupled peak and the coupled peaks, respectively (Supplementary Figures 4 and 6).

The C2UR can be grouped into three categories: low (0–15%, A, F, G, H, K, S, W, and Y), medium (20–40%, D, E, M, N, P, Q, R, and T) and high (~50%, I, L, and V) ratios (Supplementary Figure 6). The group with a low C2UR is comprised of “direct” or gluconeogenesis amino acids and the group with medium C2UR is composed of “TCA-derived” amino acids.

Note that the amino acid cysteine is absent in the primary sequence of both MBP and GB1. However, cysteine is expected to have low C2UR ratio as it is derived from serine. It should also be noted that individual amino acids in the same group also show some variation in C2UR, these variations are represented as error bars in Supplementary Figure 6 (see Supplementary Figure 7 for the complete and individual results for representative amino acid, valine, from the case of MBP). C2UR is a good indicator of which group of amino acids (direct, TCA, or ILV) a spin system originates from. Therefore, we concluded that labeling with the pre-mix strategy not only restores highly sensitive detection of HNCA resonances for every amino acid type, but also guarantees the generation of a stronger uncoupled high-resolution central peak that is devoid of coupling. In addition, the C2UR encodes auxiliary information that can be used to establish which of the 20 amino acids a spin system may be composed of.

**Fig. 4:**
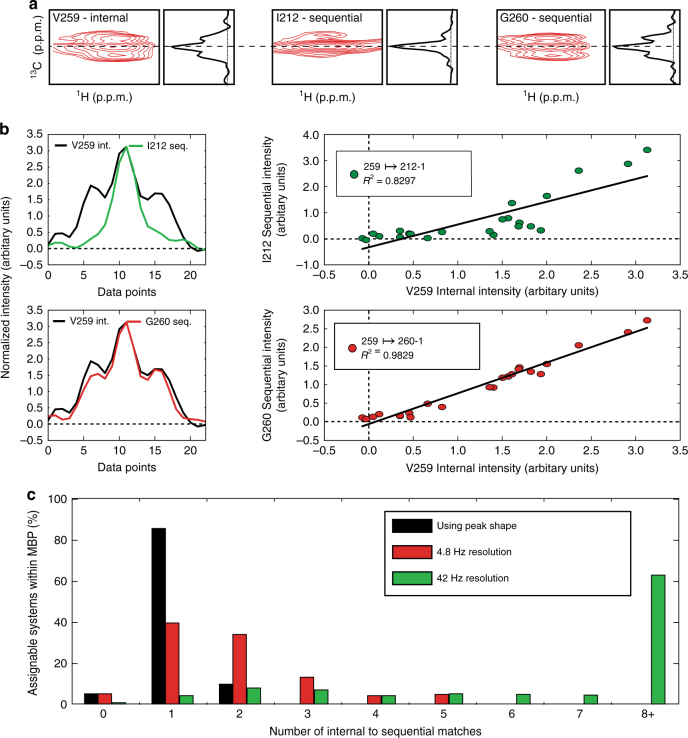
Solving the chemical shift degeneracy problem in the HNCA for the 371 amino acid protein, MBP. **a** An internal Cα peak (V259, right) and two candidate sequential peaks that match identically in chemical shift; I212 sequential (middle) and G260 sequential (left). **b** Overlay of the incorrect match (top left panel) and the correct match (bottom left panel). These matches are plotted as correlations (right panels) and scored with a correlation coefficient (inset). **c** Assignability of MBP based on chemical shift and peak shape. Completeness of assignment using a window of 42 Hz (typical HNCA, green), 4.8 Hz (high-resolution HNCA, red) and incorporation of peak matching correlations (black). Zero matches indicate that systems are adjacent to prolines or exchange broadened system

Even at this high resolution in the HNCA for MBP, there are several sequential matches for a given system based on chemical shift alone. For example, the spin system for V259 (Fig. 4a) has at least two sequential candidates that closely match in frequency space. Since the backbone assignment of MBP is known we can infer that one is the incorrect match, I212 sequential (S211), and the other is the correct match, G260 sequential. A mere visual inspection of the line shapes can unambiguously identify the correct assignment. To make this visual analysis quantitative, we overlaid intensity-normalized peaks for V259 internal and I212 sequential (Fig. 4b, top left), constructed a correlation plot of the aligned point intensities and calculated a correlational coefficient (Fig. 4b, top right). A zoom-in of this is presented in Supplementary Figure 8. Note that the intensities are normalized only for clarity in overlaying the peak and the intensity data are not normalized for the correlation plots. The same was done for the correct assignment pair, V259 internal and G260 sequential (Fig. 4b, bottom). The correct assignment showed a higher correlation coefficient, indicating that the correlation coefficient can be reliably used as a score to discriminate correct from incorrect assignments (the significance of the difference between the correlation coefficients is *p* = 0.00017). Even in cases when both the competing candidates are from the same type of amino acid, the correlation coefficient is able to establish the correct assignment (Supplementary Figure 9). In this example, subtle differences in the splitting frequency of two candidate leucines are significant enough to select the correct candidate (the significance of the difference between the correlation coefficients is *p* = 0.0013). Thus, peak shape correlation or matching proves to have the potential to make correct assignments above and beyond high resolution and C2UR.

**Fig. 5:**
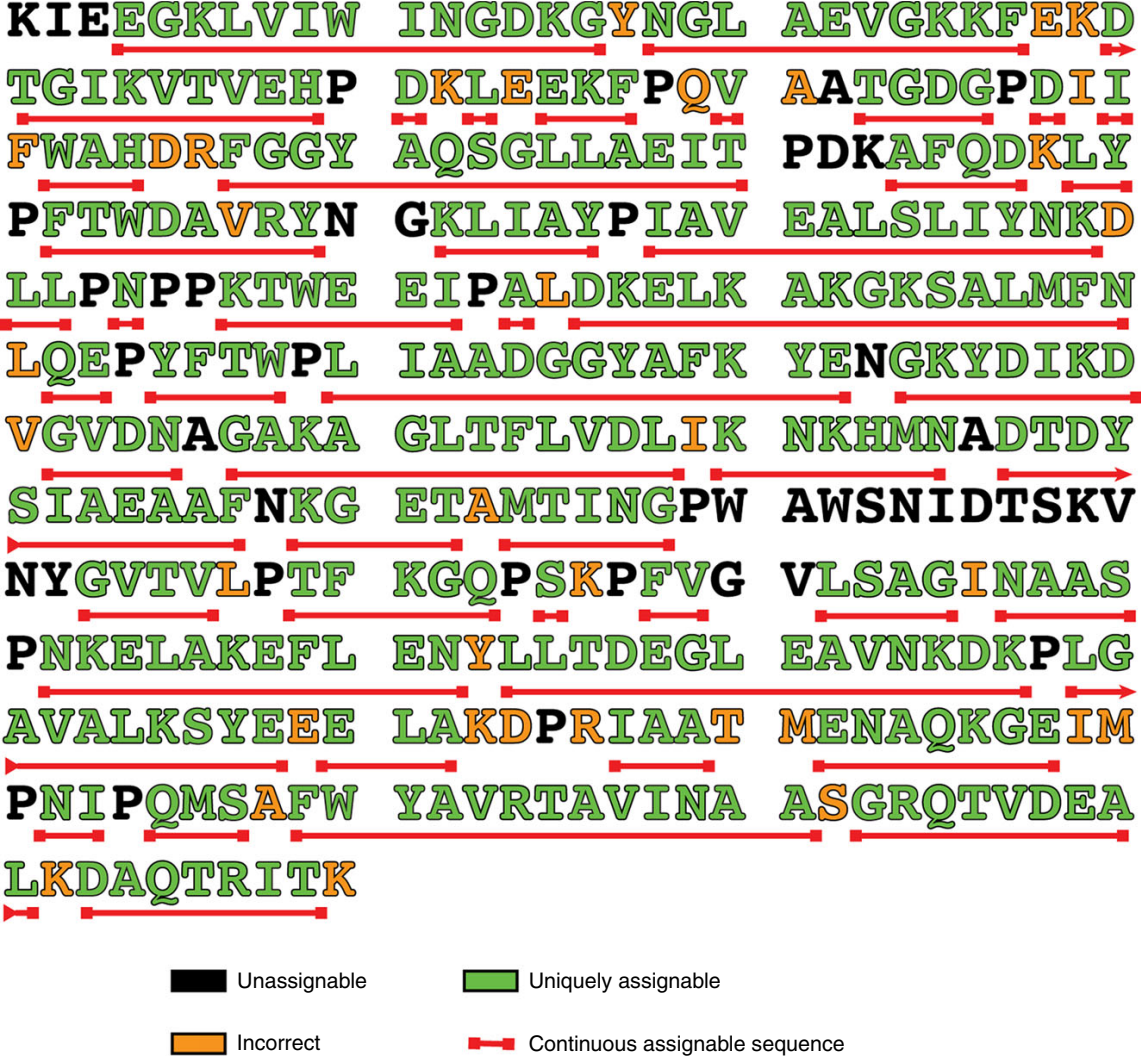
Primary structure of MBP showing the extent of assignable residues from a single HNCA experiment. Using high-resolution information along with peak correlation matching, 85% of the sequence is sequentially assignable (green). Incorrect assignments are colored orange. The long stretches of sequentially assignable sequence are underlined in red

To test the power of peak matching, we did a mock assignment of MBP using just the HNCA spectrum. If a traditional “match in frequency alone” approach is used with a resolution window of 42 Hz that corresponds to the typical resolution of HNCA for uniformly labeled sample, we can only establish unique assignment for <5% of the backbone (Fig. 4c, green). In fact, more than 60% of the residues have 8 or more matching candidates at this resolution. No more than two amino acids in a row can be sequentially assigned (Supplementary Figure 10). By increasing the resolution in the Cα dimension to 4.8 Hz, the approximate line width in our MBP spectrum, we can sequentially link about 40% of resonances by the “frequency match alone” approach (Fig. 4c, red). At this resolution, no system has more than five matching candidates for sequential connectivity. Although this is a substantial improvement, there are 200 amino acids that remain unassigned (Supplementary Figure 11). When the power of resolution is combined with the peak shape correlation analysis using the real data, over 85% of amino acids can be correctly assigned from this single HNCA experiment (Fig. 4b, black). Approximately 10% of systems cannot be uniquely assigned (Fig. 4b, black, assigned “2” to indicate degenerate matches with additional candidates). In addition, about 5% of systems cannot be assigned due to being adjacent to proline or systems that are broadened below the detection limit in our HNCA spectrum, presumably due to chemical exchange phenomena (Fig. 4b black, assigned “0” because sequential assignment is not possible). Long stretches of sequential assignment that were obtained as a result of the peak shape and peak position matching are indicated by a red bar under the sequence for MBP (Fig. 5). It should be noted that the matching algorithm employed above performs a simple pairwise matching between probable candidates and does not consider the matching probability in the context of larger stretch of the amino acid sequence. We have analyzed the cases where degeneracy remains even after peak position and peak shape matching, and the small fraction of cases where the pairwise matching apparently provide an incorrect assignment. Factors that contribute to this continued degeneracy can be classified into three categories: (1) low signal-to-noise (an example of which is discussed in Supplementary Figure 12); (2) an overlap between the internal and sequential peaks within a spin system (an example of which is discussed Supplementary Figure 13); and (3) continued degeneracy even after comparing peak shapes and peak positions (an example of which is discussed Supplementary Figure 14). Some of this can be avoided if we are able to record a spectrum with a better signal-to-noise ratio. A recent publication has shown that NUS/hmsIST can be faithfully used to extend the data set up to three times in the indirect dimension^29^. The time saved by using NUS can be devoted to more scans in the initial part of the evolution where there is more signal to improve the sensitivity. Furthermore, errors stemming from degeneracies in both peak shape and position can be reduced/eliminated if we consider sequential matches in the context of a larger stretch of the amino acid sequence. An example of this is presented in Supplementary Figure 15.

### Sensitivity of HNCA with mixed pyruvate labeling

An important aspect to consider is the difference in sensitivity in the HNCA experiment of a “mixed pyruvate” labeled sample as compared to that of a uniformly labeled sample. On average, 50% of the Cα atoms are labeled when the mixed pyruvate labeling strategy is used. In analyzing the relative sensitivity of a “mixed pyruvate” labeled sample in comparison to that of a uniformly labeled sample in the context of an out-and-back-style HNCA experiment there are two key factors that need to considered: (1) the total transfer efficiency for the N to Cα magnetization transfer and the subsequent refocusing of N with respect to Cα and (2) the gain in peak height of the central uncoupled peak in a pyruvate-labeled sample as compared to the split Cα resonance in a uniformly labeled sample. These are two independent factors and can be treated separately. A detailed analysis of this treatment is provided in Supplementary Note 1. Briefly, in the “mixed pyruvate” labeled sample there are four possible labeling combinations, (1) ^13^C(i-1)-N(i)-^13^C(i), 2) ^12^C(i-1)-N(i)-^13^C(i), 3) ^13^C(i-1)-N(i)-^12^C(i), and 4) ^12^C(i-1)-N(i)-^12^C(i), each with equal probability (*p* = 0.25) of occurrence. Note that the case where the labeling is ^13^C(i-1)-N(i)-^13^C(i) is identical to the uniform labeled sample and the case where the labeling is ^12^C(i-1)-N(i)-^12^C(i) does not yield any signal. The efficiency of transfer and transfer rates depend on the labeling pattern (Supplementary Figure 16). Combining the transfer efficiency (weighted to the probability of occurrence) and the gain in peak heights, the ratio of sensitivities of the HNCA experiment on a pyruvate-labeled sample as compared to a uniformly labeled sample is 0.6104 (ILV), 0.7181 (TCA), and 1.2207 (GNG) for 22 ms of transfer time (the case for the spectra used in this manuscript). We posit that better efficiency for mixed pyruvate samples can be achieved with transfer times closer to 42 ms. The calculations indicate sensitivity gains of 0.8695 (ILV), 1.0229 (TCA) and 1.7389 (GNG) when comparing the ratio of sensitivities of an HNCA experiment at transfer times of 42 ms and 29 ms for mixed pyruvate and uniformly labeled samples, respectively. These calculations and associated sensitivity gains account for relaxation losses during the transfer time for the protein MBP (42 kDa) and are summarized in Supplementary Table 1. To experimentally verify the sensitivity gains of the pyruvate sample described above, we acquired ^1^HN/^13^C 2D planes from a TROSY-HNCA experiment with an array of transfer times between 18 and 90 ms on a mixed pyruvate-labeled, perdeuterated GB1 sample. The 2D planes contained sufficient resolution to determine peak heights for many spin systems. The plot of peak intensity for spin system F54 as a function of transfer time both for the internal (red solid line) and sequential (black solid line) is shown in Supplementary Figure 17. We then modeled our calculated sensitivity equations for internal and sequential peaks based on J_NC_ couplings of 11.0 and 8.2 Hz for the internal and sequential peaks, respectively, and a relaxation rate (R_2_) of 4 Hz. Our calculations for internal (dashed red) and sequential (dashed black) systems predict the data quite well. It should be noted that optimal transfer times for each residue will vary from the calculated value, depending on the individual relaxation time and if the residue experiences exchange broadening.

Since the method described here relies on matching the high-resolution central frequency and the residue specific peak shape information, fidelity in encoding these two components is important. To obtain high-resolution in the Cα dimension the protein should be expressed in its deuterated form, as Cα atoms attached to hydrogen experience faster relaxation. Cα attached to deuterium relaxes ~8 times slower than those attached to hydrogen, which allows longer acquisition times. However, one drawback of deuteration is the incomplete back exchange of the observable amide hydrogens. In some proteins, this incomplete back exchange will result in two populations of amides N–H and N–D. Although there is no magnetization that can start or end with the N–D species, it will create an isotope shift on the Cα resonance^30^. In a case where a residue i-1 harbors an amide that is incompletely exchanged, the amide of the sequential residue i will encode the weighted sum of two frequencies, each corresponding to the Cα attached to the N–H and N–D, respectively (Supplementary Figure 18). However, the amide of residue i-1 will only encode the frequency corresponding to the Cα attached to N–H. Thus, the match in peak positions will not be ideal This is discussed further in Supplementary Note 2. Effort should be taken to maximize the back exchange of amides to hydrogen. Several established approaches have been used to maximize the back exchange. These include equilibrating the sample at basic pH (~8–9), equilibrating the sample at high temperature, refolding and partial unfolding with refolding. A detailed explanation of the effect of incomplete back exchange is provided in the Supplemental Information.

## Discussion

The use of pyruvate as a carbon source for protein expression has been exploited previously for generating ^1^H-^13^C methyl groups^31,32^ and (^1^Hα)^13^Cα-^13^CO spin-pairs^33^ in an otherwise deuterated background. The labeling scheme described here is not a mere extension of the 1-^13^C-labeled or 2-^13^C-labeled glucose or SAIL strategies. The “mixed pyruvate” labeling strategy opens a new avenue for backbone assignment. This labeling strategy provides two independent sets of information (1) high-resolution chemical shift information and (2) unique peak shape from residual carbon ^1^J_αβ_ couplings, which are amino acid specific. The combination provides site-specific information beyond the resolution of amino acid type. The labeling strategy would not only be useful in backbone resonance assignment but could also be expanded to establish unambiguously assigned NOEs, particularly for ^15^N-dispersed NOESY experiments.

Here we have used deuterated 2-^13^C-labeled and 3-^13^C-labeled pyruvate in various combinations to label GB1 and MBP (Supplementary Table 2) with the aim to explore labeling patterns and improve relaxation properties for backbone assignment purposes. The pre-mix labeling strategy allows unique simultaneous access to ultra-high-resolution peaks, amino acid type classification via the C2UR and peak shape correlation tests between candidate systems that enables backbone assignments of large protein systems using the sensitive HNCA experiment alone. The 10% of systems that were incorrectly sequentially aligned when using the intersection of frequency and peak shape can be potentially corrected when attempting to match the C2UR ratios of aligned systems to the amino acid sequence. We anticipate this match will form part of a future automatic assignment procedure. This model could be further expanded to delineate redundant matches that persist. For instance, if there is degeneracy where both the uncoupled center frequency and the correlations are a close match, one could use selective narrow band Cβ decoupling during the Cα evolution period in an additional HNCA experiment. This will collapse the side lobes for the resonances that have a Cβ chemical shift matched with the decoupled frequency. In addition, ^13^C incorporation at the CO position can be also harnessed to augment peak shapes if the Cα-CO coupling is permitted to partly evolve during Cα encoding. The details of the labeling pattern at the CO position, both in the presence of ^13^C and ^12^C carbonate, needs to be explored in a separate study. Furthermore, the ^1^JC_α_C_β_ couplings provided in the spectrum harbor vital information about the local backbone conformation^34^. Although we have not specifically harnessed this aspect in this study, the dependence of the coupling on the backbone geometry could aid the matching process, especially to differentiate degeneracy involving the same amino acid type.

Based on list prices, the cost of making a mixed pyruvate sample grown with 1.5 grams of 2-^13^C and 1.5 grams of 3-^13^C pyruvate is ~13.5% more expensive when compared to the traditional uniformly labeled sample cultured with 3 g of ^13^C-deuterated glucose. It should be noted that the pyruvate used is purchased as a protonated molecule and is easily exchanged to a deuterated form as described in the Experimental Methods section. In addition, if the demand for protonated ^13^C pyruvate goes up, the price of labeled pyruvate should decrease, thus making the price of the mixed pyruvate sample comparable or cheaper to the perdeuterated, uniformly labeled sample. Although the effect of induction lengths and varying ratios of 2-^13^C/3-^13^C pyruvates in expression media on the subsequent labeling pattern could benefit from further optimization, the pre-mix strategy, as currently implemented, will nevertheless yield ultra-sharp uncoupled resonances and amino acid dependent peak shapes, which extends the resolution and the power of resonance differentiation beyond what has been previously possible. This unique feature will allow us to push the limit of backbone resonance assignment for larger biological systems.

## Methods

### Preparation of growth media with deuterated pyruvate

3 grams of 2-^13^C and/or 3-^13^C pyruvate were dissolved in 1 kg of D_2_O (99.9%) and the pH of the solution was adjusted to ~13.0 by 2.5 mM NaOD to deuterate the methyl protons of pyruvate by proton–deuteron exchange. The exchange was carried out for 30 min at room temperature with occasional shaking. pH was restored to neutral (pH 7.0) by the addition of 4.26 g Na_2_HPO_4_, 3.60 g NaH_2_PO_4_, and 3.00 g KH_2_PO_4_. The media were also supplemented with 1.0 g of ^15^N or ^14^N ammonium chloride depending on desired labeling state for nitrogen and 1.0 g of ^12^C sodium bicarbonate. The media also contained 0.24 g of MgSO_4_, 0.011 g CaCl_2_ and necessary antibiotics (50 mg kanamycin, 50 mg carbenicillin or 100 mg ampicillin). The media was sterilized through filtration (0.22 μM) before use.

### Culture conditions and sample preparation

A single colony of expression strain (GB1 or MBP) was grown overnight at 37 °C in 10 mL of filter sterilized LB media with 100% D_2_O substituted for water. The cells were pelleted and resuspended in 10 mL of the deuterated pyruvate media and allowed to grow for 6–8 h at 37 °C. These cells were again pelleted and resuspended in 900 mL of the deuterated pyruvate media and allowed to grow at 37 °C overnight. When the OD at 600 nm reached a value between 0.4 and 0.6, which occurs 16–24 h later, the temperature was decreased to 20 °C and cells where induced by addition of 1 mM IPTG. The proteins were expressed for an additional 24 h. Cells were harvested by centrifugation and protein was purified through standard affinity purification procedures. Briefly, cells expressing GB1 were lysed by sonication in lysis buffer (50 mM Tris-HCl, 150 mM NaCl, pH 8.0). The lysate was purified by a single pass over Ni-NTA resin, which was washed with three column volumes of lysis buffer and eluted with 300 mM imidazole, 50 mM Tris, 150 mM NaCl pH 8.0 buffer. Protein was buffer exchanged into 50 mM sodium phosphate, 50 mM NaCl pH 6.5. SDS–PAGE established the sample was sufficiently pure for NMR analysis. The protein was concentrated to 1 mM for NMR analysis. MBP protein was purified after sonication in lysis buffer (50 mM Tris-HCl, 150 mM NaCl, pH 8.0) by passing the lysate over immobilized amylose beads and eluted with 10 mM maltose in 50 mM Tris-HCl, pH 8.0. The elution was buffer exchanged into 10 mM HEPES, 1 mM EDTA, pH 6.5. The amides of MBP were back exchanged by addition of 1 M urea to this buffer and allowing exchange to take place at 37 °C for 24 h, followed by a buffer exchange back to NMR buffer (10 mM HEPES, 1 mM EDTA, pH 6.5). MBP was concentrated to 600 μM and β-cyclodextrin was added to the sample to a final concentration of 2 mM.

### NMR spectra

NMR data collection of GB1 samples was performed on a Bruker 750 MHz instrument equipped with a cryogenically cooled probe. The TROSY-HNCA pulse sequence from the standard Bruker library was used with deuterium decoupling. Sweep widths for the ^1^H, ^15^N and ^13^C dimension were 10,504, 2430, and 6031 Hz, respectively. The indirect dimensions were sampled non-uniformly, selecting 2500 out of a Nyquist grid of 54 × 512 (^15^N × ^13^C) complex points (~9% sampling). Sampling schedule was chosen based on the Poisson gap sine weighted protocol^16^. Data collection on the MBP sample was performed on a Bruker 900 MHz instrument equipped with a cryogenically cooled probe. The standard Bruker TROSY-HNCA pulse sequence (trhncagp2h3d2)^35^ was used with modification to permit non-uniform sampling with Topspin 2.1. Sweep widths for the ^1^H, ^15^N, and ^13^C dimension were 12,626, 3375, and 7243 Hz, respectively. The indirect dimensions were sampled non-uniformly, selecting 5216 out of a matrix of 75 × 750 (^15^N × ^13^C) complex points (~9% sampling). Sampling schedule was selected based on the Poisson gap sine weighted protocol. See Supplementary Methods for more details.

### NMR data reconstruction

NUS spectra were reconstructed using the hmsIST software package and NMRPipe^36^. 400 iterations of iterative soft threshold (IST) reconstruction were used^16^. Each dimension was zero filled before regular Fourier transformation. For GB1, this results in 1024 real points in the ^13^Cα dimension for a sweep width of 6031 Hz, or a digital resolution of 5.9 Hz. For MBP, a zero fill to 2048 points was used giving a digital resolution of 3.5 Hz. However, a cosine window function was applied to the 750 complex points before Fourier transformation, which made the effective digital resolution ~4.8 Hz.

### NMR data extraction and analysis

Data was analyzed using purpose-built Python software and chemical shifts from the Biological Magnetic Resonance Data Bank (BMRB). Specifically, NMRPipe format spectra were directly read and 1D traces along the ^13^C dimension of systems were extracted based on chemical shifts reported in the BMRB^13^ (Entry # 7114). It was necessary to make minor adjustments to the reported BMRB chemical shifts to account for changes that arose from differences in sample preparation and experimental conditions. Coupled to uncoupled peak height ratios (C2UR) were calculated after fitting of peaks to a “three- peak” model. Specifically, the Cα peak intensity at a point in the spectrum (*x*) was modeled as being composed of three Gaussian peaks; a central uncoupled peak (centered at *m*) and assumed to be of largest height (*k*_1_), and two equally sized (height *k*_2_) and spaced coupled peaks (distance *d* from the central peak) on either side of the central peak. Line width (*s*) was assumed to be equal for all peaks. Thus, the following equation (1) was derived for the three-peak model:

1 $${\rm Intensity}\left( x \right) = k_2 * \mathrm{e}^{ - \frac{{\left( {x - m - d} \right)^2}}{{2s^2}}} + k_1 * \mathrm{e}^{ - \frac{{\left( {x - m} \right)^2}}{{2s^2}}} + k_2 * \mathrm{e}^{ - \frac{{\left( {x - m + d} \right)^2}}{{2s^2}}}$$

Data exceeding the width of a peak (~80 Hz, or 33 points for MBP HNCA spectrum) was considered for fitting. A curve fit function written in Python was used. The parameters *k*_1_ and *k*_2_ were used to estimate the peak heights of the uncoupled central peak and the coupled adjacent peaks. The mock assignment procedure was performed by first declaring an assignment as being unambiguous if, based on the known assignments from the BMRB, there was one and only one match between an internal chemical shift in a spin system and a sequential chemical shift in another spin system within a “resolution” window. Resolution limits of 42 Hz (regular HNCA resolution) and 4.8 Hz (high-resolution HNCA) were then used. Then a check that the correct internal-sequential match was done. Afterwards, all remaining ambiguous matches were examined for peak shape matching by performing a correlation coefficient calculation between an internal peak and all frequency matching sequential peaks within the resolution window. In this case, 23 spectral points were used. The highest correlation was declared the best match and then verified based on known assignments from the BMRB. Correct and incorrect assignments after pattern matching were tallied. In addition, systems that could not be sequentially assigned due to non-existent sequential systems that are missing (i.e., proline or exchange broadened systems) were counted separately.

Additional details on the methods used are provided in the Supplementary Methods section of the Supplementary Material.

### Data availability

Other data are available from the corresponding author upon reasonable request.

## Electronic supplementary material

Supplementary Information
