# Supplementary material for: Mixed pyruvate labeling enables backbone resonance assignment of large proteins using a single experiment
Source: Nat Commun. 2018 Jan 24;9:356. doi: 10.1038/s41467-017-02767-8 (PMC5783931; doi:10.1038/s41467-017-02767-8)
Supplement: Supplementary file 1 — Supplementary Information [file 41467_2017_2767_MOESM1_ESM.pdf]

Mixed pyruvate labeling enables backbone resonance assignment of large proteins using a single experiment

Robson et al

Supplementary Figures

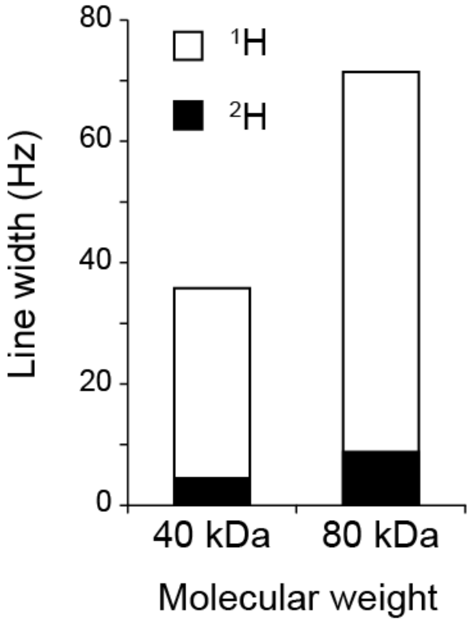

Supplementary Figure 1: Estimated line widths of $^{13}C\alpha$ from a 40 kDa and an 80 kDa protein at 800 MHz. Left and right columns are for a 40 kDa and an 80 kDa protein respectively, at 298K in H_2O buffer. The estimated line widths of deuterated and non-deuterated proteins are shown with filled and open bars, respectively. The linewidths for the $^{13}C\alpha$ in a deuterated protein would be 4.4 Hz for a 40 kDa protein and 8.8 Hz for an 80 kDa protein. Here dipolar contribution from the remote atoms ($^{1}H\beta$ and ^{1}HN in the same residue), neighboring $^{13}C\beta$, $^{13}C'$ and ^{15}N, and CSA are taken into account. The distance of $^{1}H\beta$ and ^{1}HN to $^{13}C\alpha$ was set to 2.16 Å and 2.24 Å, respectively. Dipolar contributions from two $^{1}H\beta$ hydrogen atoms were considered as this is representative of the majority of the amino acids. The difference of the two principal components of the axially symmetric $^{13}C\alpha$ chemical shift tensor was set to 21.5 ppm. The difference in the bond length between $^{1}H\alpha$-$^{13}C\alpha$ (1.09 Å) and $^{2}H\alpha$-$^{13}C\alpha$ (1.05 Å) was also taken into account.

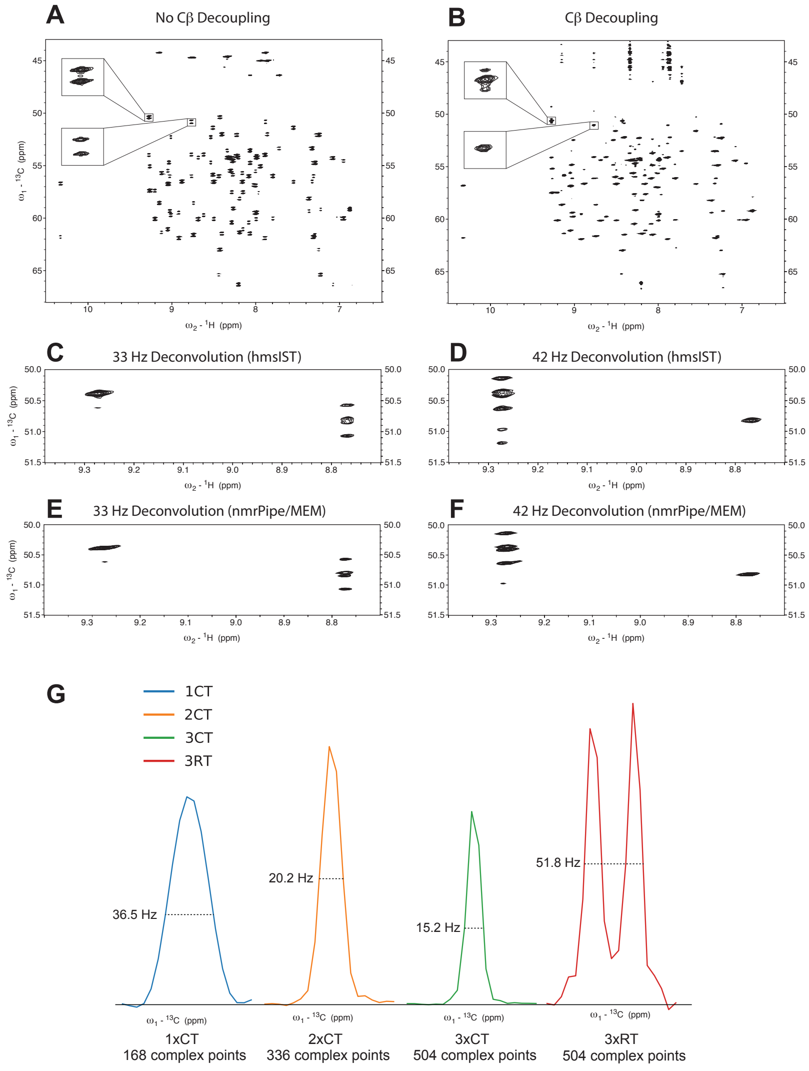

Supplementary Figure 2: Effect of $^{1}J_{\alpha\beta}$ to the ^{13}Cα line shape in uniformly ^{13}C labeled protein and established methods to remove the coupling. (A and B) A 2D HNCA plane of uniformly ^{13}C-labeled GB1 (A) without and (B) with band-selective $^{13}C_{\beta}$ decoupling during ^{13}Cα evolution. (A) All Cα signals show peak splitting due to the ^{13}Cα-^{13}Cβ couplings, apart from glycine at ~45 ppm. The ^{13}Cα-^{13}Cβ coupling constants are not identical (compare top peak inset box to bottom peak inset box). (B) With ^{13}Cβ decoupling, most of the ^{13}Cα-^{13}Cβ couplings are decoupled, but not all (top peak inset box). The line shapes of glycine resonances at the edge of the decoupling bandwidth (~45 ppm) are distorted. (C-F) Virtual decoupling was performed post-acquisition, to remove the $^{1}J_{\alpha\beta}$ coupling and representative resonances that are in the inset of panel A, are shown in panel

C, D, E and F. In the case of C and D the deconvolution was performed by the hmsIST method corresponding to coupling constants of 35 Hz and 42 Hz, respectively. In panel E and F, the ME deconvolution method in nmrPipe was applied assuming coupling constants of 35 Hz and 42 Hz, respectively. The virtual decoupling was achieved by applying the deconvolution frequencies along the ^{13}Cα dimension. However, no single decoupling frequency removes the $^{1}J_{\alpha\beta}$ coupling across the entire spectrum, most likely due to variation in the magnitude of the couplings that are present in the spectrum (35-42 Hz). (G) 1D traces from an HNCA of GB1 through the internal Cα peak along the ^{13}C dimension of residue F54. The $^{1}J_{\alpha\beta}$ coupling is removed by evolving the Cα dimension in a constant time (CT) fashion. 1x, 2x and 3xCT periods (26.6 ms, 53.2 ms and 79.8 ms, respectively) and a real-time evolution equivalent to 3xCT periods (real time or RT) were compared. Half-height linewidths from nmrPipe estimation are indicated. Note that the linewidth for the RT peak was estimated manually as nmrPipe could not model this peak. Elongation of the CT periods improve resolution; however, there is significant loss in sensitivity, despite the longer acquisition time for the 3xCT spectrum, even for small proteins, such as GB1 (6 kDa). This loss in sensitivity would be prohibitive for large systems. These data cumulatively show that current decoupling technologies are insufficient to fully suppress the $^{1}J_{\alpha\beta}$ coupling.

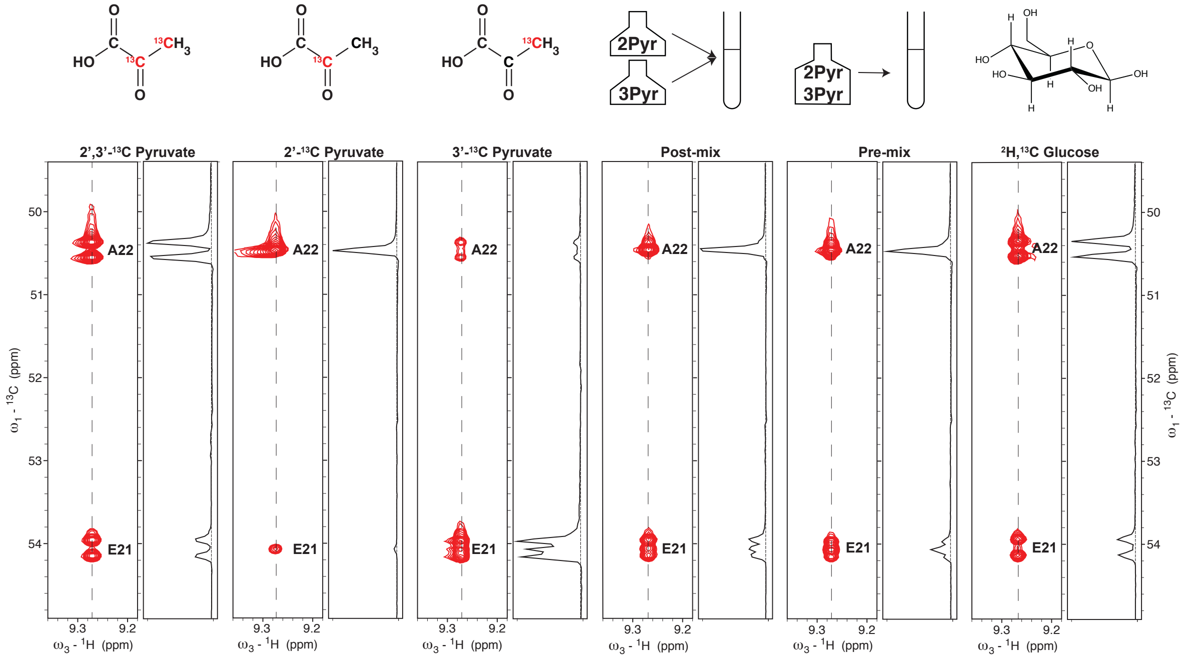

Supplementary Figure 3: Labeling pattern at Cα with various carbon sources and mixtures for the A22 spin system in GB1. Displayed here are 2D strips from a 3D HNCA experiment corresponding to residue A22. The labeling scheme for each spectrum is indicated at the top of each strip with the chemical structure or schematic of labeling. A 1D trace is plotted next to each strip.

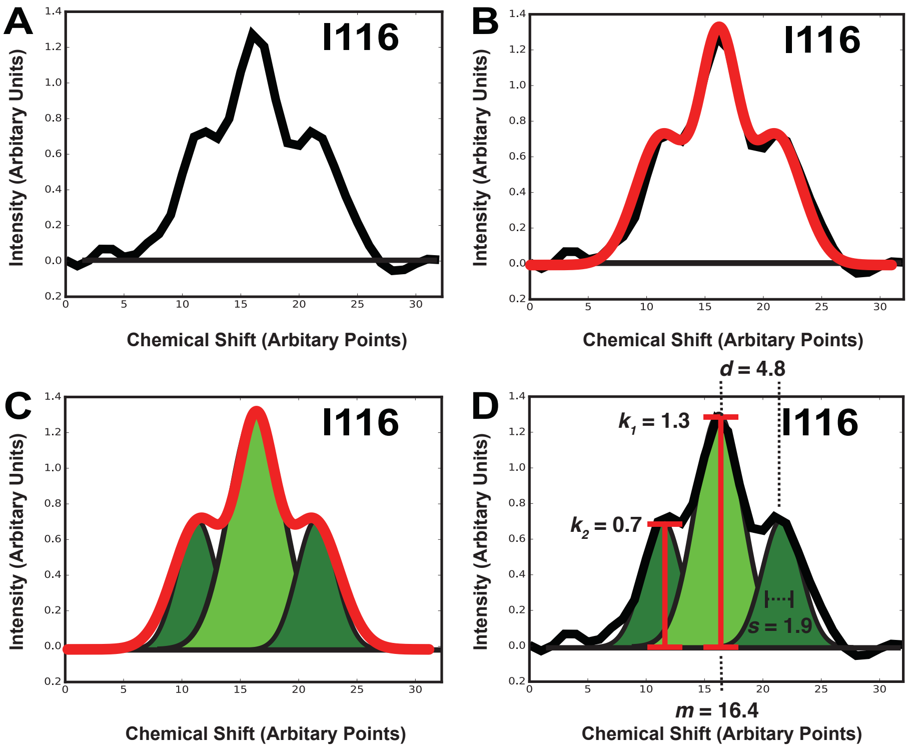

Supplementary Figure 4: Example of fitting the ratios of coupled and uncoupled resonances to extract quantitative metrics. Internal peak from system I116 was modeled as a 'three peak' system (See "NMR Data Extraction and Analysis" in the main text). (A) The raw intensity data. (B) A plot of the fitted peak (red) overlaid on the raw intensity data. (C) Independently plotted deconvoluted peaks. The coupled peaks (dark green) and the uncoupled peak (light green) with the combined model plotted as a red line. D) Fitted parameters from the equation in "NMR Data Extraction and Analysis" are shown. The coupled to uncoupled peak height ratio (**C2UR**) is calculated as the ratio k_2/k_1.

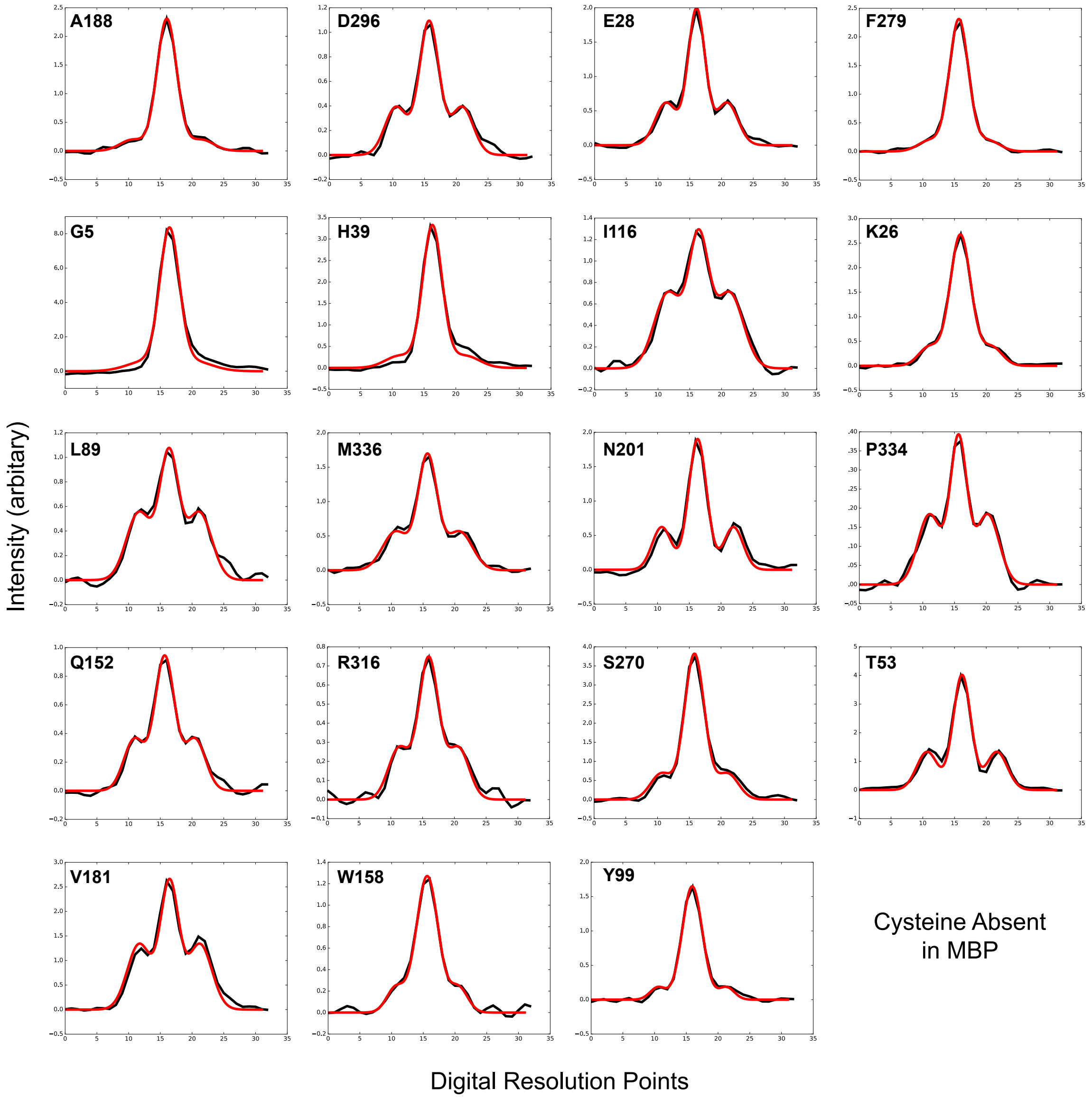

Supplementary Figure 5: Representative peak shapes of the Cα resonances for the various amino acids as result of 2-^{13}C and 3-^{13}C premix labeling. The Cα resonances from the 19 amino acids present in MBP (cysteine is absent). Each amino acid has a predominant, central, uncoupled peak. The raw data (black lines) were successfully fitted with a three-peak model (red line); see Supplementary Figure 4 and "NMR Data Extraction and Analysis" in main text.

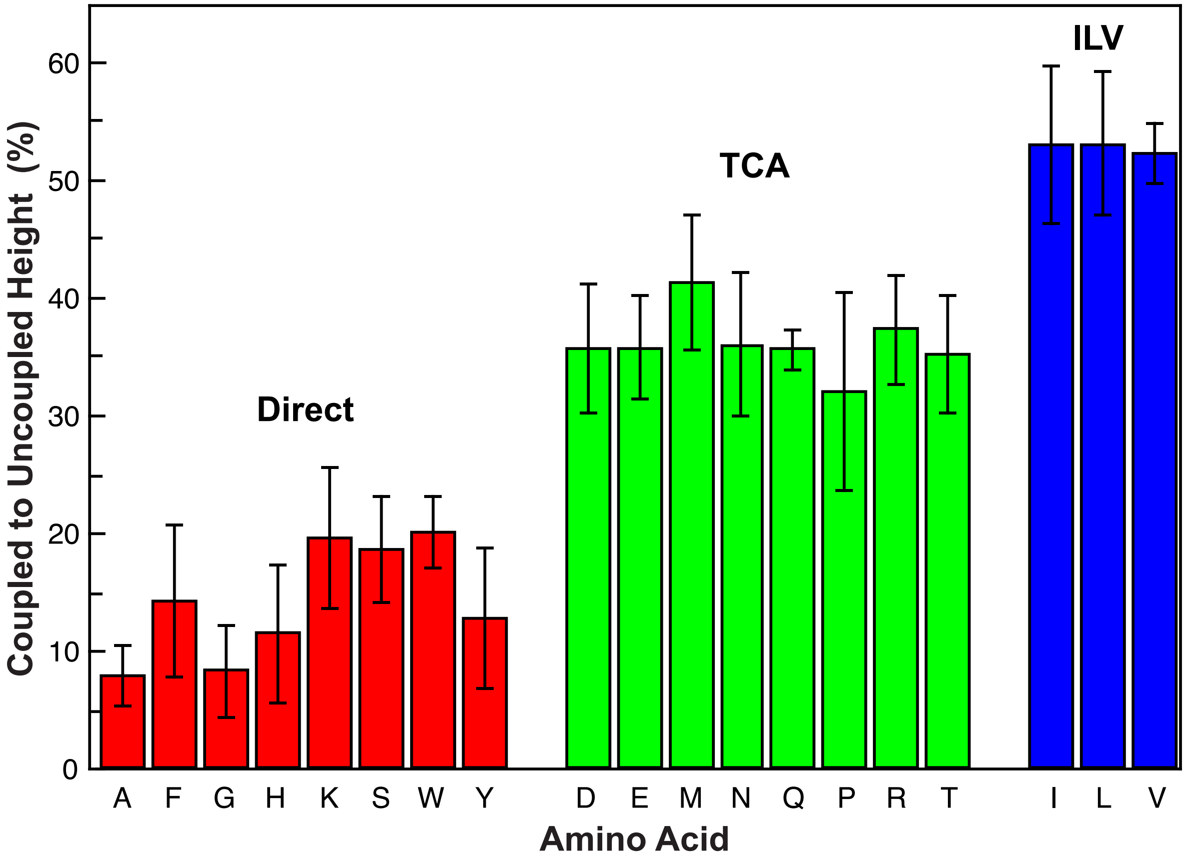

Supplementary Figure 6: Bar graph of the coupled to uncoupled peak height ratio (C2UR) for each amino acid type grouped by the metabolic pathway from which they are derived. The ratio was calculated from the fitting with a three-peak model (See Supplementary Figure 4 and "NMR Data Extraction and Analysis" in the main text). For each amino acid, the error bar represents the standard deviation of the C2UR ratio calculated from all the occurrences of the amino acid in MBP.

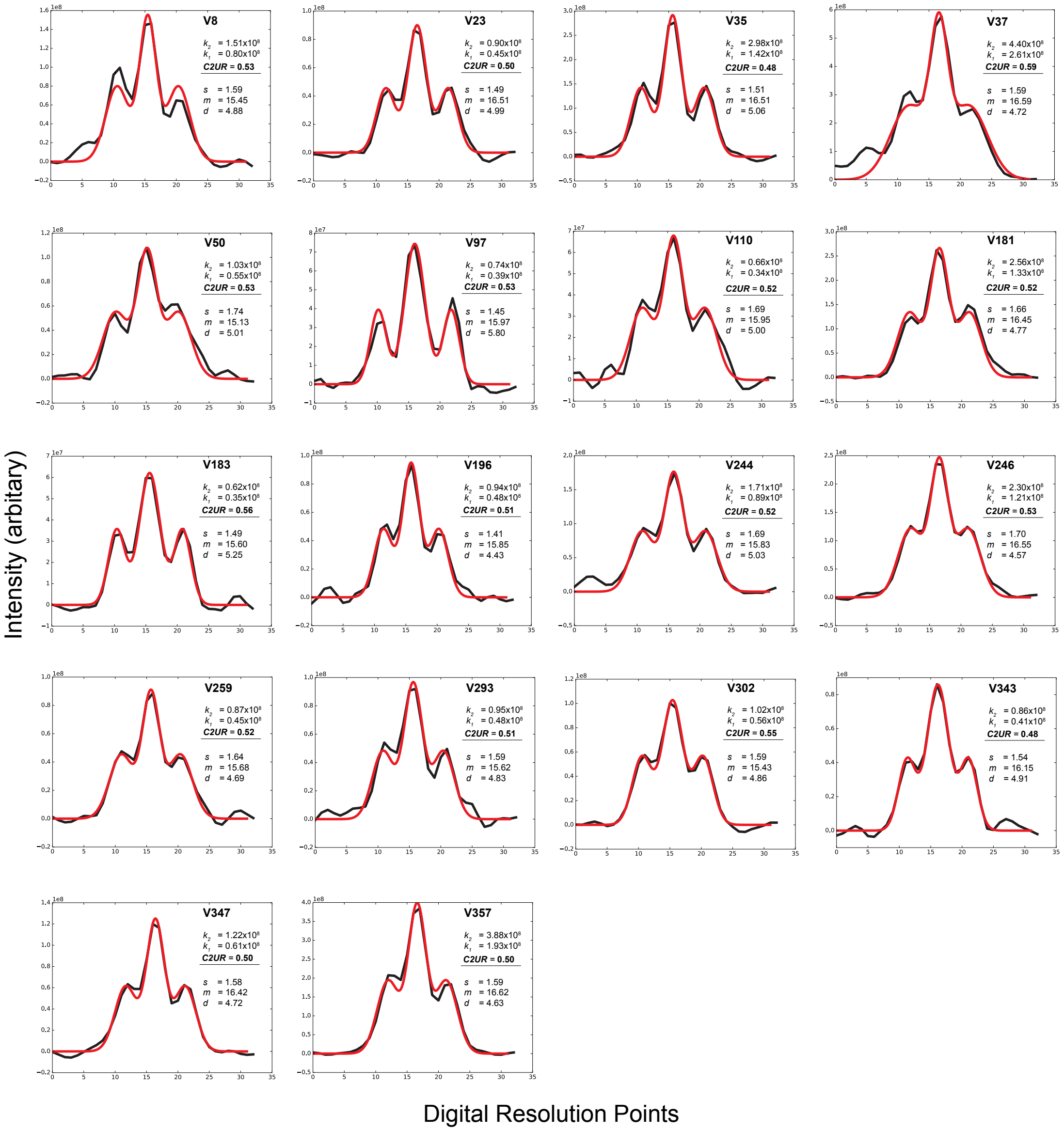

Supplementary Figure 7: Complete set of peak fittings for all valine residues in MBP. Three peak fitting parameters are given for each valine along with their calculated C2UR. The raw data (black lines) were successfully fitted with a three-peak model (red line). Although these amino acids are taken from the same metabolic pool with C2UR values around 0.5, there is still variability in this calculation, some of which is attributable to the signal to noise of the individual peak. Note that there is considerable variability in the distance between the uncoupled peak and one of the coupled peaks (determined as the value 'd' in units of points in the spectrum), with a range of 4.43 to 5.80.

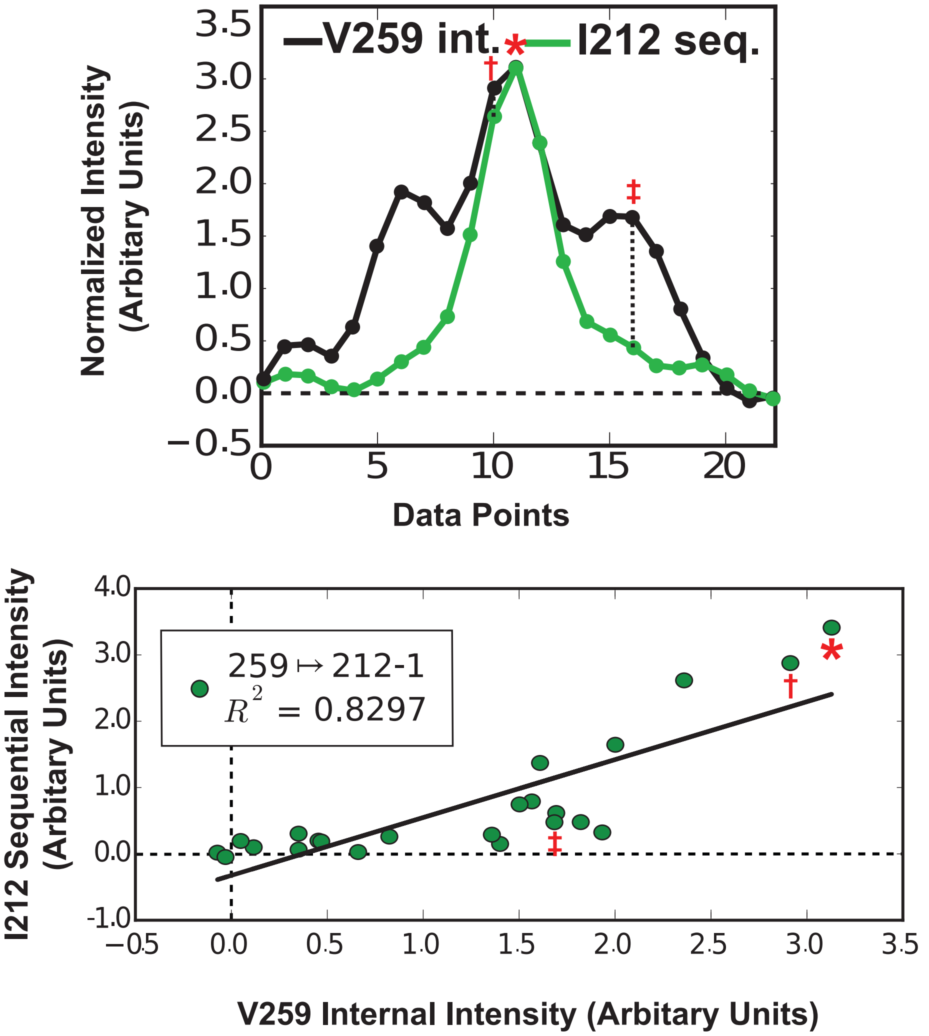

Supplementary Figure 8: Zoom in plot of Figure 4B, top panel with markers added for clarity. Top) The incorrect sequential peak, I212 (green line), is overlaid with the V259 (black line) internal peak and digital points (green and black, respectively) added so the frequency match, by point, can be seen. Three corresponding points are highlighted and indicated with symbols *, † and ‡. Points at * and † correspond closely and contribute to a high correlation. Points at ‡ have a large mismatch and contribute to a poor correlation. Bottom) Frequency points from I212 sequential and V259 internal are plotted on a correlation plot. The solid line is the line of best fit of all the points.

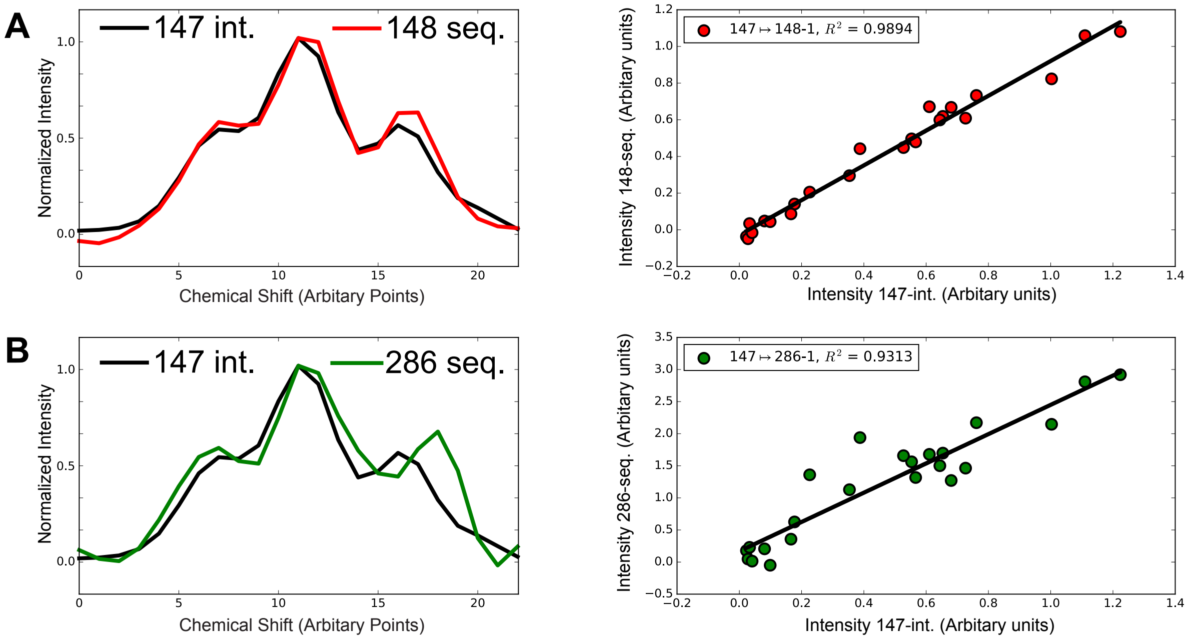

Supplementary Figure 9: Using correlation coefficient to discriminate between the correct and incorrect assignment when the matching candidates are the same amino acid type. Overlay of (A) the correct sequential candidate (148 sequential) and (B) the incorrect sequential candidate (286 sequential) with the L147 internal peak signal. The internal peaks are shown in black and those of the matched sequential candidates are shown in color (red and green). Both candidates show the same chemical shift and have leucine as the sequential residue. The peak shape correlation coefficient is higher for A than for B (A; R^2 = 0.9894, B; R^2 = 0.9313; p = 0.0013), enabling successful discrimination of the right candidate despite both having the same amino acid type (Leu). This is confirmed by visual examination of the peak shape, which shows that the coupled peak spread is wider for L285 than for L147 due to a larger $^{13}C\alpha^{13}C\beta$ coupling.

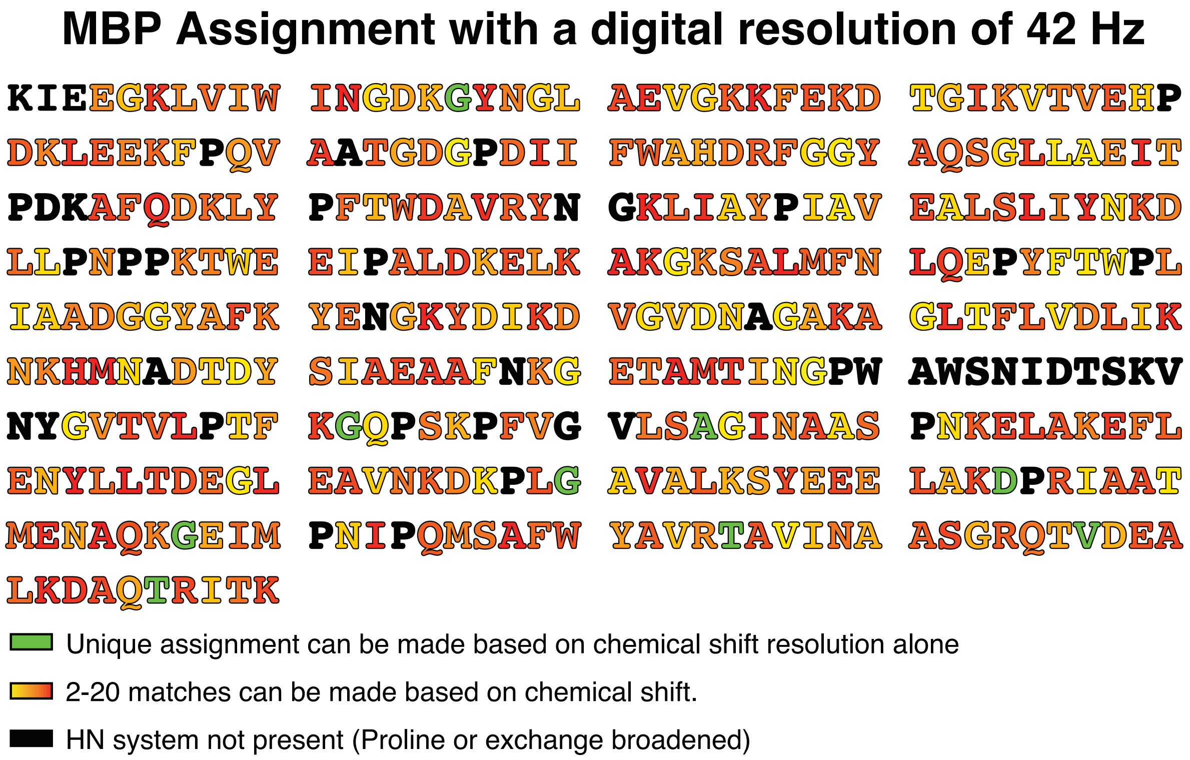

Supplementary Figure 10: Assignability of MBP using the HNCA with regular resolution (42 Hz). Amino acid sequence of MBP is color coded by the level of assignability using the HNCA alone at a Cα resolution of 42 Hz. Only ~5% of amino acids (green) can be uniquely matched to a sequential partner. Other amino acids cannot be unambiguously assigned. The number of possible candidates for a sequential match, based on frequency alignment, for each residue is represented as a heat map. The maximum number of match possibilities for any given residue is 20 (see legend).

MBP Assignment with a digital resolution of 4.8 Hz

KIEEGKLVIW INGDKGYNGL AEVGKKFEKD TGIKVTVEHP
DKLEEKFPQV AATGDGPDII FWAHDRFGGY AQSGLLAEIT
PDKAFQDKLY PFTWDAVRYN GKLIAYPIAV EALSLIYNKD
LLPNPPKTWE EIPALDKELK AKGKSALMFN LQEPYFTWPL
IAADGGYAFK YENGKYDIKD VGVDNAGAKA GLTFLVDLIK
NKHMNADTDY SIAEAAFNKG ETAMTINGPW AWSNIDTSKV
NYGVTVLPTF KGQPSKPFVG VLSAGINAAS PNKELAKEFL
ENYLLTDEGL EAVNKDKPLG AVALKSYEEE LAKDPRIAAT
MENAQKGEIM PNIPQMSAFW YAVRTAVINA ASGRQTVDEA
LKDAQTRITK

- Unique assignment can be made based on chemical shift resolution alone
- 2-5 matches can be made based on chemical shift.
- HN system not present (Proline or exchange broadened)

Supplementary Figure 11: Assignability of MBP using the HNCA with higher resolution (4.8 Hz). Primary amino acid sequence of MBP is color coded by the level of assignability using the HNCA alone at a Cα resolution of 4.8 Hz. Increasing resolution to 4.8 Hz improves the number of unique sequential matches to ~40% (green). The number of match possibilities for sequential assignment is represented as a heat map as in Supplementary Figure 10. The maximum number of overlapping matches was reduced to 5.

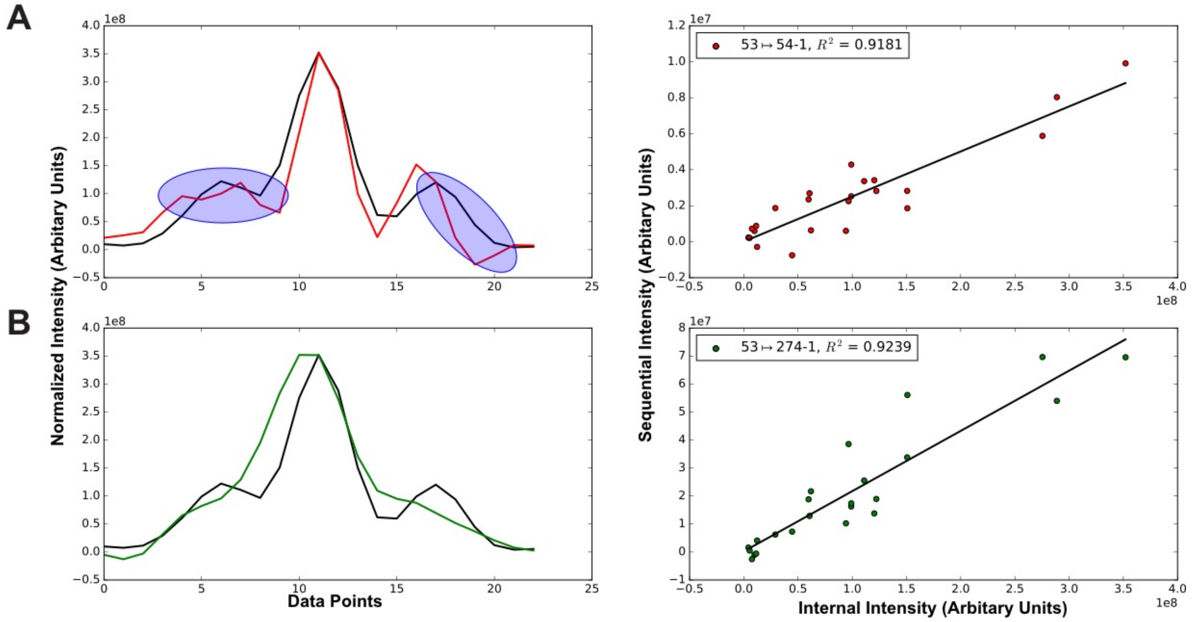

Supplementary Figure 12: Example of a plausible incorrect assignment match due to interference from noise. Example of a case where inadequate signal to noise affects the match. In this case, we are looking for a match for the internal peak of residue 53, represented in black. The Cα-1 peak for amino acid 54 (Panel A, red line), which should be the correct match, has apparent mismatches in peak shape when compared to the internal peak (Panel A, black line), as indicated by the blue ovals, due to the low signal to noise for the Cα-1 peak. These deviations (blue ovals) result in a diminished correlation coefficient (R^2 = 0.9181) when compared to that for the amino acid 274 Cα-1 peak (Panel B, green line, R^2 = 0.9239), leading to an incorrect assignment when pairwise matching is done. This can be remedied by increasing the signal-to-noise ratio and/or considering the match in the context of a larger fragment of the primary sequence (example shown in Supplementary Figure 14).

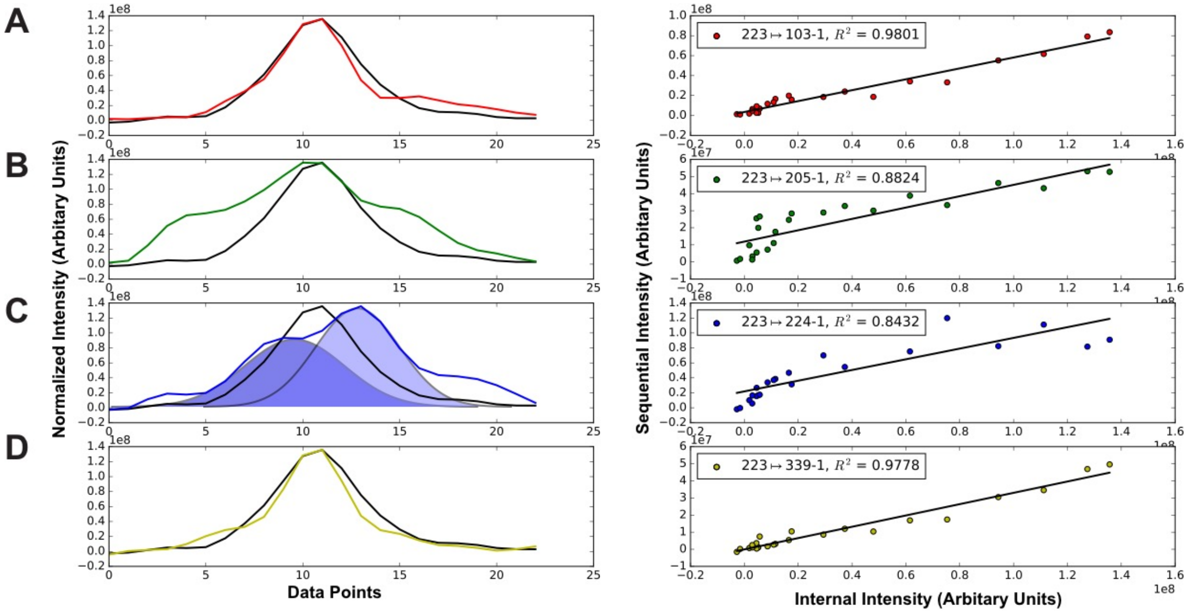

Supplementary Figure 13: Example of degeneracy due to overlap in sequential/internal peaks. In this case, we are looking for a match for the internal peak of residue 223, represented in black. The correct sequential candidate would be the Cα-1 for amino acid 224 (Panel C, darker blue peak), which does not match the peak for Cα 223. This is because there is an overlap in frequency between the internal and sequential resonances of residue 224. The frequency of the internal Cα resonance is just upfield (Panel C, lighter blue peak). This overlap reduces the overall R^2 value to 0.8432, below that of other systems: 0.9801 (Panel A), 0.8824 (Panel B) and 0.9778 (Panel D). This type of error can be rectified by considering the match in the context of a larger fragment of the primary sequence (example shown in Supplementary Figure 15).

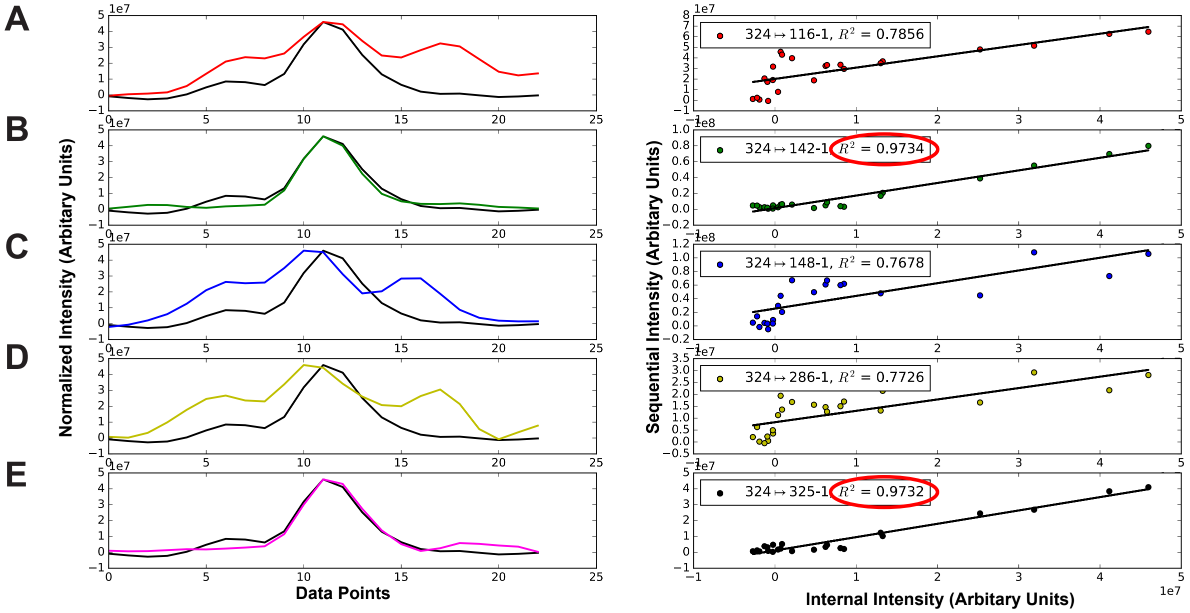

Supplementary Figure 14: Example continued degeneracy after comparing peak positions and peak shapes. In this example, we are looking for a match for the internal peak of residue 324, represented in black. The correlations of sequential candidates shown in Panels A, C and D are poor and therefore eliminate them as probable candidates. However, panel B with the incorrect assignment (R^2 = 0.9734) shows a slightly better correlation than panel E with the correct assignment (R^2 = 0.9732). This is a case where there is a degeneracy in both peak position and peak shapes. Selective Cβ decoupling (discussed in the main manuscript) could break this degeneracy.

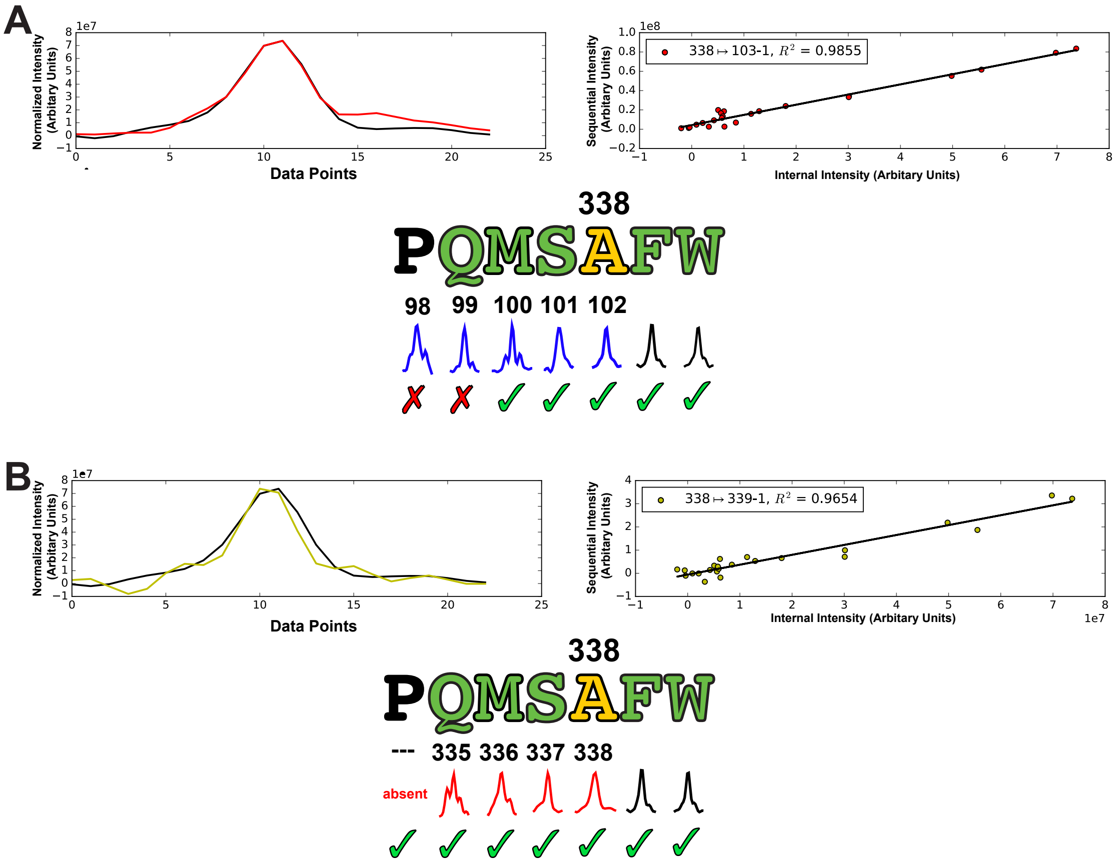

Supplementary Figure 15: Example of breaking continued degeneracy and incorrect assignment correction by matching Cα peak shapes in the context of a larger stretch of primary amino acid sequence. Panel A contains a better correlation (R^2 = 0.9855) than panel B (R^2 = 0.9654) and is presumed to be the correct assignment; however, this assignment is incorrect based on previous knowledge. If we align the resultant Cα peaks to the amino acid sequence (Panel A, Blue peaks under the sequence), we find that after a few sequential spin systems are added we begin to see discordance between the known sequence and the spin systems. Specifically, the peak shape for Q335 is not typical for a glutamine and we find a sequential alignment to another spin system where there should be none (a proline). On the other hand, the discounted, but correct, assignment in panel B leads to a sequence of Cα peaks that do match the primary sequence (Panel B, red peaks). Specifically, Q335 has the correct peak shape and our failure to align a spin system to the N-terminus of Q335 agrees with the expectation of a proline at this position.

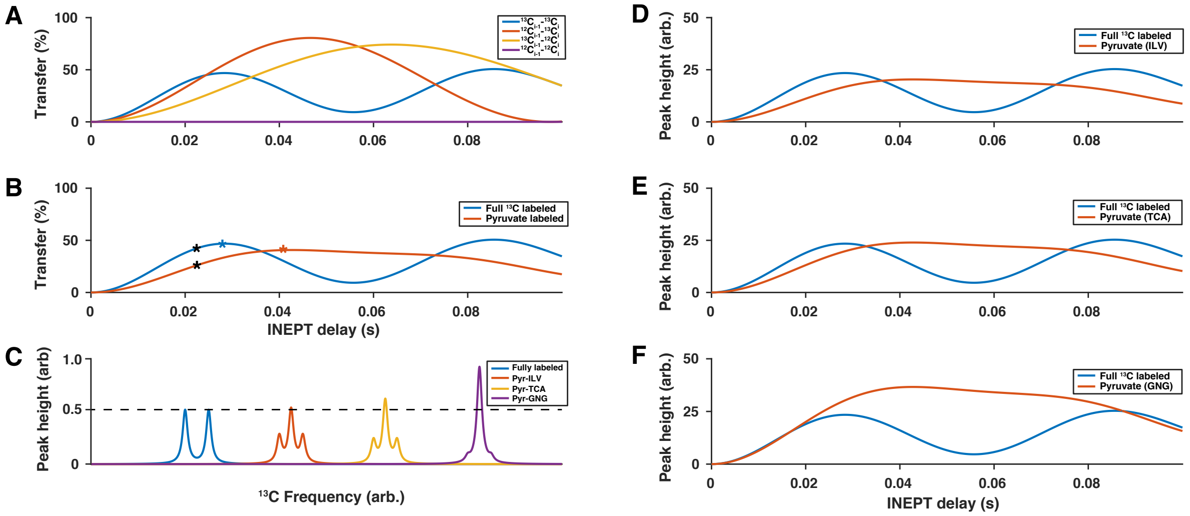

Supplementary Figure 16: Transfer efficiencies and relative sensitivity in mixed pyruvate and uniformly labeled sample. A: The total transfer efficiency for the N to Cα magnetization transfer (N_y ->$N_zC^{\alpha}_z$) and the subsequent refocusing of N with respect to Cα ($N_yC^{\alpha}_z$ -> N_y) for four different labeling scenarios: 1) $^{13}C_{(i-1)}$-$N_{(i)}$-$^{13}C_{(i)}$ (blue), 2) $^{12}C_{(i-1)}$-$N_{(i)}$-$^{13}C_{(i)}$ (red), 3) $^{13}C_{(i-1)}$-$N_{(i)}$-$^{12}C_{(i)}$ (yellow), and 4) $^{12}C_{(i-1)}$-$N_{(i)}$-$^{12}C_{(i)}$ (purple). These curves are calculated for the protein MBP with a ^{15}N-TROSY relaxation time (T_2) of 220 ms. B) The combined and weighted transfer efficiency for the pyruvate and the uniformly labeled sample. The black asterisks (*) indicate the conventionally used transfer time of 22ms. The blue asterisk (*) indicates an optimal transfer time of 29 ms for a uniformly ^{13}C-labeled sample of MBP. The brown asterisk (*) indicates an optimal transfer time of 42 ms for a “pre-mix” pyruvate ^{13}C-labeled sample of MBP. C) Representative Cα peak shapes for a uniformly labeled sample (blue), ILV residues from a “pre-mix” pyruvate sample (brown), amino acids derived through the TCA cycle from a “pre-mix” pyruvate sample (yellow), and amino acids derived through the GNG cycle from a “pre-mix” pyruvate sample (purple). D), E), and F) Sensitivity gains (represented in terms of peak height) by combining contributions of transfer efficiency and lack of Cα-Cβ splitting in a “pre-mix” pyruvate sample for the three different pathways by which amino acids are generated in “pre-mix” pyruvate labeling scheme.

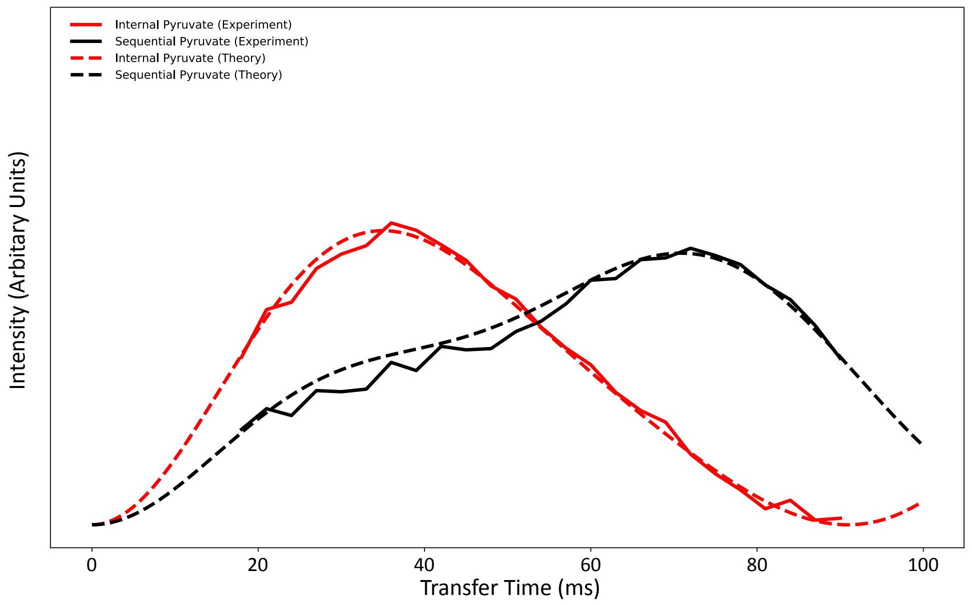

Supplementary Figure 17: Transfer Time versus Intensity (internal and sequential) for theoretical and experimental results for GB1. Peaks heights from ^{1}H-^{13}C 2D planes of an HNCA as a function of transfer delays used for N_y ->$N_zC^{\alpha}{}_z$ (and back) are plotted for the internal peak (solid red line) and the sequential peak (solid black line) for F54 in GB1. Using J_{NC} couplings of 11.0 and 8.2 Hz for internal and sequential peaks and an R_2 of 4 Hz with our above transfer equations resulted in a model (dashed red line for internal and dashed black line for sequential peaks, respectively) that matched the data well. Note that the J_{NC} couplings used are typical of β-sheet secondary structure and F54 in GB1 is in β-sheet space.

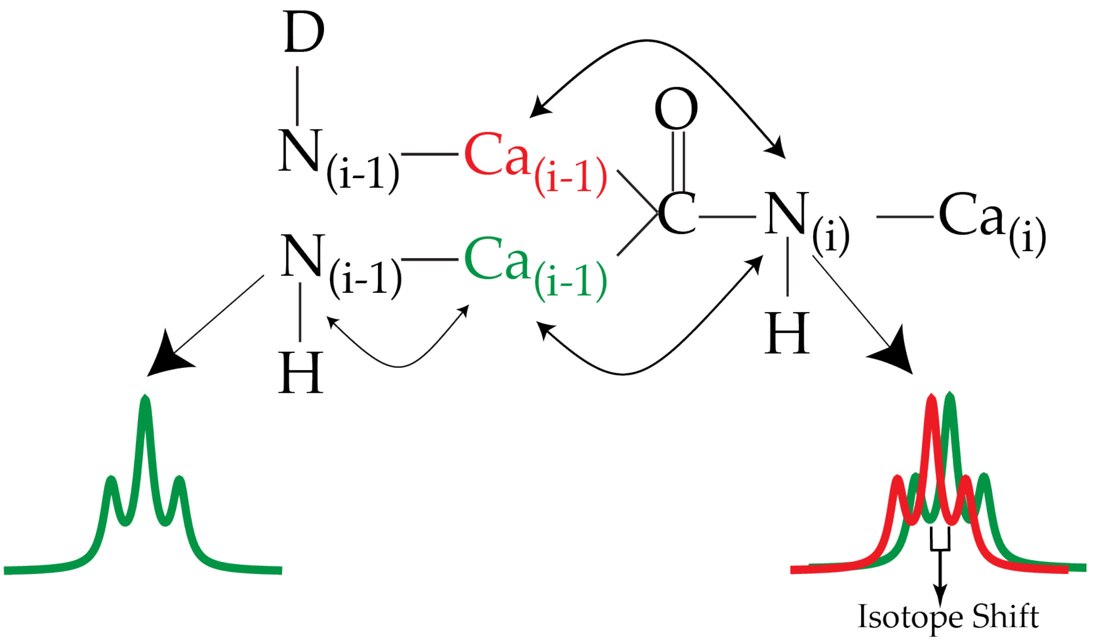

Supplementary Figure 18: Graphical representation of magnetization transfer between N and Cα in an HNCA experiment. In this case, the amide of residue i-1 is incompletely back-exchanged and exists in two different forms N-D and N-H. Red peaks and green peaks correspond to the Cα frequency where the corresponding amide nitrogen (N(i-1) in this case) is N-D or N-H respectively.

Supplementary Tables

Supplementary Table 1: Summary of theoretical sensitivity changes between fully-labeled and "pre-mix" pyruvate samples. The values shown here are the ratio of the sensitivities of a TROSY-HNCA experiment on a mixed pyruvate sample to those of a uniformly labeled MBP sample. Relaxation losses during the transfer delays have been accounted for.

Amino Acid Type	Sensitivity (Premix / Fully Labeled) (delay = 22 ms)	Sensitivity (Premix / Fully Labeled) (Optimal versus standard delay (42 v 29 ms))
**ILV**	0.6104	0.8695
**TCA**	0.7181	1.0229
**GNG**	1.2207	1.7389

Supplementary Table 2: Summary of samples and the labeling pattern used in this study

Index	Protein	Type of Labeling	Referred to as:
**1**	GB1 (8 kDa)	^{2}H-^{13}C glucose	Fully Labeled GB1
**2**	GB1 (8 kDa)	2-^{13}C pyruvate	2-^{13}C pyruvate Labeled GB1
**3**	GB1 (8 kDa)	3-^{13}C pyruvate	3-^{13}C pyruvate Labeled GB1
**4**	GB1 (8 kDa)	1:1 ratio of **2** and **3**	"Post-mix" GB1
**5**	GB1 (8 kDa)	1:1 ratio 2- and 3-^{13}C pyruvate	"Pre-mix" GB1
**6**	MBP (42 kDa)	1:1 ratio 2- and 3-^{13}C pyruvate	"Pre-mix" MBP

Supplementary Note 1

Sensitivity of an HNCA experiment on a "mixed pyruvate" labeled sample as compared to that of a uniformly labeled sample

On average, 50% of the Cα atoms are labeled using the mixed pyruvate strategy. In analyzing the relative sensitivity of a "pre-mix pyruvate" labeled sample in comparison to a uniformly labeled sample in an out-and-back style HNCA experiment there are two key factors that need to considered: 1) the total transfer efficiency for the N-to-Cα magnetization transfer (N_y ->$N_zC^{\alpha}_z$) and the subsequent refocusing of N with respect to Cα ($N_yC^{\alpha}_z$ -> N_y), and 2) the gain in peak height of the central uncoupled peak in a pyruvate labeled sample as compared to the split Cα resonance in a uniformly labeled sample. These are two independent factors and will be discussed separately. The remainder of the pulse sequence behaves identically between the uniformly and the pyruvate-labeled samples in terms of transfer efficiency, relaxation, and sensitivity, except for the small, but still significant difference in relaxation during the Cα evolution. This difference is due to the absence of ^{13}C-labeled Cβ, which works in favor of the "premix pyruvate" labeled sample. It should be noted that this affects only the central uncoupled peak and that this benefit is amino-acid dependent. When considering the absence of ^{13}C-labeled Cβ during the Cα evolution, the T2 is extended by ~14 % for MBP (τ_c = 20ns) at 800 MHz.

Different transfer efficiency for the N-to-Cα magnetization transfer (N_y -> $N_zC^{\alpha}_z$) and refocusing of N with respect to Cα ($N_yC^{\alpha}_z$ -> N_y)

In a uniformly labeled sample all Cα carbons are ^{13}C-labeled and therefore, with respect to any given amide pair, the internal and sequential Cα are 100% ^{13}C-labeled. In the "mixed pyruvate" labeled sample there are four possibilities: 1) $^{13}C_{(i-1)}$-$N_{(i)}$-$^{13}C_{(i)}$, 2) $^{12}C_{(i-1)}$-$N_{(i)}$-$^{13}C_{(i)}$, 3) $^{13}C_{(i-1)}$-$N_{(i)}$-$^{12}C_{(i)}$, and 4) $^{12}C_{(i-1)}$-$N_{(i)}$-$^{12}C_{(i)}$, each with equal probability (p=0.25) of occurring. Note that the case where the labeling is $^{13}C_{(i-1)}$-$N_{(i)}$-$^{13}C_{(i)}$ is identical to the situation in a uniformly labeled sample, and the case where the labeling is $^{12}C_{(i-1)}$-$N_{(i)}$-$^{12}C_{(i)}$ does not yield any signal. The out-and-back transfer efficiency for each of the four cases, including accounting for relaxation losses during the transfer, are given below by the following transfer functions, where t is the INEPT transfer time, T2 is the relaxation of ^{15}N (the TROSY component of ^{15}N in this case), and J_{seq} (10.25 Hz) and J_{intra}

(7.35 Hz) are the coupling constants of the backbone amide nitrogen to the sequential and internal Cα carbons respectively:

Case 1: $^{13}C_{(i-1)}$-$^{15}N_{(i)}$-$^{13}C_{(i)}$ (this is also the case for a uniformly labeled sample)

Transfer Efficiency to Sequential:

$[\exp(-t/T_2) \times \sin(\pi J_{seq} \cdot t) \times \cos(\pi J_{intra} \cdot t)] \times [\exp(-t/T_2) \times \sin(\pi J_{seq} \cdot t) \times \cos(\pi J_{intra} \cdot t)]$

Transfer Efficiency to Internal:

$[\exp(-t/T_2) \times \sin(\pi J_{intra} \cdot t) \times \cos(\pi J_{seq} \cdot t)] \times [\exp(-t/T_2) \times \sin(\pi J_{intra} \cdot t) \times \cos(\pi J_{seq} \cdot t)]$

Case 2: $^{12}C_{(i-1)}$-$^{15}N_{(i)}$-$^{13}C_{(i)}$

Transfer Efficiency to Sequential:

NIL

Transfer Efficiency to Internal:

$[\exp(-t/T_2) \times \sin(\pi J_{intra} \cdot t)] \times [\exp(-t/T_2) \times \sin(\pi J_{intra} \cdot t)]$

Case 3: $^{13}C_{(i-1)}$- $^{15}N_{(i)}$-$^{12}C_{(i)}$

Transfer efficiency to Sequential:

$[\exp(-t/T_2) \times \sin(\pi J_{seq} \cdot t)] \times [\exp(-t/T_2) \times \sin(\pi J_{seq} \cdot t)]$

Transfer efficiency to Internal:

NIL

Case 4: $^{12}C_{(i-1)}$- $^{15}N_{(i)}$-$^{12}C_{(i)}$

Transfer efficiency to Sequential:

NIL

Transfer efficiency to Internal:

NIL

The efficiency of transfer and the buildup is different in each case and is plotted in Supplementary Figure 16, Panel A. The efficiency for the uniformly labeled sample is calculated as the sum of the internal and sequential efficiency over various transfer times (blue). For cases 2 and 3, where only one of the Cα is labeled, the transfer maximizes around $1/2J_{intra}$ (Case 2, red) and $1/2J_{seq}$ (Case 3, yellow) respectively. For Case 4, no transfer is possible and is indicated with a purple line at 0. Therefore, for a pyruvate-labeled sample, the total efficiency is calculated as the weighted sum of the four cases with equal weighting of 0.25 corresponding to their respective probabilities. This is seen in Supplementary Figure 16, Panel B. Thus, the transfer efficiency, when using "pre-mix" pyruvate labeling can be maximized by the appropriate choice of transfer times. Since the TROSY component of ^{15}N relaxes slowly and is not greatly affected by the molecular weight of the system, there will only be a marginal loss in applying longer transfer times to large molecular weight systems. Relaxation was accounted for in the above calculations by using a T2 of 220 ms, which corresponds to the ^{15}N-TROSY relaxation of MBP. In addition, the^{15}N-TROSY relaxation time can be extended by using higher magnetic fields (900-1000 MHz)[1].

Based on these theoretical values, the optimal transfer time for the "mixed pyruvate" MBP sample is ~42 ms (brown asterisk, Panel B). This contrasts with the transfer time for fully labeled samples, which has a theoretical maximum at ~29 ms (blue asterisk). Conventionally, spectra are acquired with a standard 22 ms delay, which is indicated with the black asterisks.

Gain in peak height of the central uncoupled peak in a premix pyruvate labeled sample compared to that of the split CA resonance in a uniformly labeled sample.

In practice, the sensitivity of an NMR experiment is determined by the ratio of the peak height to the noise level. In the case of the pyruvate labeling scheme the major central peak has reduced or absent Cβ coupling and is expected to have up to twice the peak height as compared to that of a uniformly labeled sample. In practice, this gain in the peak height depends on the percentage of ^{13}Cα carbons at a given residue position that are not adjacent to a ^{13}C-labeled Cβ atom. This in turn depends on the type of amino acid and the pathway by which they are derived (see Figure 1 and Supplementary Figure 6). In Supplementary Figure 16, Panel C, we have shown model peak shapes for fully labeled (blue),

Pyruvate-ILV (red), Pyruvate-TCA (yellow) and Pyruvate-GNG (purple; gluconeogenesis) to indicate how each of the three types of peak shapes seen in our data would have improved signal height over a fully labeled sample because of the high-resolution spectra. In Panels D-F we have plotted the signal heights against increasing delay time for ILV, TCA and GNG, respectively, in comparison to a fully labeled sample. Combining the transfer efficiency and the gain in peak heights, the ratio in sensitivity of the HNCA experiment on either the ILV, TCA, or GNG amino acids in a pyruvate labeled sample to that of the uniformly labeled sample is 0.6104 (ILV), 0.7181 (TCA) and 1.2207 (GNG) for 22 ms of transfer time (as used for the spectra referenced here). We theorize that better efficiency for pyruvate samples can be achieved with transfer times closer to 42 ms. Our calculations suggest sensitivity gains of 0.8695 (ILV), 1.0229 (TCA) and 1.7389 (GNG) when comparing delay times of 42 ms for pyruvate samples and 22 ms for fully labeled samples. These calculations and associated sensitivity gains account for relaxation losses during the transfer period for the protein MBP (42 kDa), and are summarized in Supplementary Table 1.

Supplementary Note 2

Importance of maximizing the back-exchange of amide protons

In cases where protein is expressed in D_2O and there is incomplete back-exchange of the deuterium attached to the backbone amide (N-D) to hydrogen (N-H) during the protein purification process, the 2-bond and 3-bond isotope shift of D on the Cα resonance will affect the matching procedure described here. Effort should be made to maximize back-exchange of the amides to N-H. Several approaches have already been established to encourage back-exchange. These include equilibrating the sample at basic pH (~8 to 9), equilibrating the sample at high temperature, refolding, and partial unfolding and refolding [2-3]. We would like to note the samples discussed in this study are fully back-exchanged.

The 2-bond isotope shift on Cα ($^{2}\Delta C_{\alpha}$) is ~18 Hz and the 3-bond isotope shift ($^{3}\Delta C^{\alpha}$) varies between 2 and 10 Hz depending on the secondary structure[4]. If the residue i-1 is incompletely back-exchanged, there will be two species (N-D) and (N-H) with two different C^{α}_{i-1} resonances, $C^{\alpha}_{i-1}(H)$ and $C^{\alpha}_{i-1}(D)$. The internal transfer of magnetization from

the amide N_{i-1} will encode only the $C^{\alpha}_{i-1}(H)$ frequency, because there will be no magnetization transfer from the N-D species. Whereas, the sequential transfer of magnetization from the amide residue i (N_i) will be transferred to both $C^{\alpha}_{i-1}(H)$ and $C^{\alpha}_{i-1}(D)$. Thus, a weighted sum (based on the populations of N-H and N-D species) of $C^{\alpha}_{i-1}(H)$ and $C^{\alpha}_{i-1}(D)$ frequencies, with their respective isotopic shifts will be encoded by the amide residue N_i. This will degrade the correlation between the internal and sequential peaks. In a case where the ith residue is also incompletely back-exchanged, a relatively smaller 3-bond isotope shift will be encoded only by the amide of the $i\text{-}1^{th}$ residue. The magnitude of these shifts depends on the secondary structure context of the relevant amino acids. This isotope shift is not ideal for peak shape matching and care should be taken to obtain maximum back exchange to N-H. A graphical representation of this is shown in Supplementary Figure 18.

Supplementary Methods

Deuteration of Commercially Purchased Pyruvate

All protons of 2-^{13}C and 3-^{13}C pyruvate were exchanged to deuterons by dissolving up to 3 grams of the relevant pyruvate or pyruvate mix into 1 kg of D_2O (99.9%). The pH of the solution was adjusted to ~13.0 by addition of NaOD to a final concentration of 2.5 mM. Specifically, 278 μL of 8.1 M NaOD was added to approximately 900 mL of D_2O. This mixture was allowed to sit with occasional shaking for at least 30 minutes to permit exchange to take place. Neutral pH was restored by the addition of anhydrous phosphate buffer components as dry powder (see below).

Preparation of Growth Medium

Growth medium was prepared after pyruvate exchange in the same 1 kg of D_2O. The following components were added in the order stated here.

The following phosphate components were added to the 1 kg of D_2O to bring the solution back to neutral pH:

- 4.26 g Na_2HPO_4 anhydrous
- 3.60 g NaH_2PO_4 anhydrous
- 3.00 g KH_2PO_4 anhydrous

The solution was shaken for approximately five minutes until all the components were completely dissolved.

1.0 g $^{15}NH_4Cl$ (if isotopic labeling of nitrogen is desired) or $^{14}NH_4Cl$ was then added and allowed to dissolve completely.

Then 1.0 g of non-isotopically enriched $NaHCO_3$ was added and allowed to dissolve completely.

0.24 g of anhydrous $MgSO_4$ was added and completely dissolved, followed by:

- 50 uL of vitamin mix

- 500 uL of trace elements, dehydrated and resuspended in 500 uL of the medium prepared so far.

The requisite amount of antibiotic for 1 L (e.g. 50 mg kanamycin, 50 mg carbenicillin, or 100 mg ampicillin) was added as dry powder, followed by 0.011 g anhydrous $CaCl_2$. Please note that the anhydrous $CaCl_2$ may not completely dissolve.

Finally, the media was filter-sterilized before being ready for use.

Cell Culture Conditions

A single colony of GB1 or MBP expression strain (T7 Express Competent *E. coli*, New England Biolabs) was grown in 10 mL of filter-sterilized LB culture made up in 99.9% D_2O overnight at 37 ºC. The next day, these cells were pelleted by centrifugation and the media aspirated. The cells were then resuspended in 10 mL of the deuterated pyruvate media as prepared above and allowed to grow for 6-8 hours at 37 ºC. These cells were again pelleted and resuspended in another 10 mL of the pyruvate media. This suspension was then immediately added to the remainder of the media and allowed to grow at 37 ºC overnight. Culture growth is slower than that of cultures grown with ^{13}C-^{2}H glucose for uniformly labelled samples and therefore there is little concern it will 'overgrow' overnight. The following day, the optical density (OD) at a wavelength of 600 nm was monitored and cells were induced at an OD between 0.4 and 0.6, which typically occurs 10-16 hours later. Upon reaching the desired OD range, the incubation temperature was dropped to 20 ºC and protein expression was induced by addition of ~250 mg of powdered IPTG to a final concentration ~1 mM. Cells were incubated with shaking for an additional 24 hours following the addition of IPTG. Final cell densities are typically below an OD_{600} of 1.0. Cells were then harvested and protein purified using standard protocols. Despite the low cell density, the yields were all approximately 10 mg of protein / L of culture.

Back-exchange of Amides

As both GB1 and MBP were expressed in D_2O, back-exchange of all amide hydrogens to protons is necessary for successful triple resonance experiments. We found amide hydrogen exchange in GB1 was sufficient following incubation of the protein in its final protonated NMR buffer (50 mM Na_3PO_4, 50 mM NaCl, pH 6.5) for 24 hours at 37 ºC. MBP was back-exchanged by the addition of 1M urea to its NMR buffer (10 mM HEPES, 1 mM EDTA, pH 6.5) followed by incubation at 37 ºC for 24 hours. The sample was then buffer exchanged by centrifugal ultrafiltration (EDM Millipore) back into its NMR buffer.

NMR Data Collection

Data collection on GB1 samples (1 mM) was performed on a Bruker 750 MHz instrument with cryogenically cooled probe (TCI). The pulse sequence was the standard Bruker TROSY-HNCA[5] in Topspin 3.1. Sweep widths for the ^{1}H, ^{15}N, and ^{13}C dimension were 10504, 2430, and 6031 Hz respectively. The indirect dimensions were sampled non-uniformly, with 2500 complex points selected from a Nyquist grid of 54 by 512 (^{15}N x ^{13}C), or a total of 27648, complex points (approximately 9% sampling). The sampling schedules were selected based on the Poisson Gap Sine Weighted protocol[6]. 8 scans were collected for each point with a recycle delay of 1 second, giving a total acquisition time of approximately 22 hours. Data collection on the MBP sample (600 uM) was performed on a Bruker 900 MHz instrument with cryogenically cooled probe (TCI). The pulse sequence was the standard Bruker TROSY-HNCA in Topspin 2.1 (trhncagp2h3d2), with the phase acquisition portion of the pulse program edited to permit non-uniform sampling. Sweep widths for the ^{1}H, ^{15}N, and ^{13}C dimension were 12626, 3375, and 7243 Hz respectively. The indirect dimensions were sampled non-uniformly, with 5216 complex points selected from a Nyquist grid of 75 by 750 (^{15}N x ^{13}C), or a total of 56250, complex points (approximately 9% sampling). The sampling schedules were selected based on the Poisson Gap Sine Weighted protocol. 16 scans were collected for each point with a recycle delay of 1 second, giving a total collection time of ~4.2 days.

NMR Data Reconstruction

Spectra were reconstructed using the hmsIST software package and nmrPipe[7]. 400 iterations of iterative soft threshold (IST) reconstruction were used. Each dimension was zero filled before Fourier transformation. For GB1, this results in 1024 real points in the ^{13}Cα dimension for a sweep width of 6031 Hz, or a digital resolution of 5.9 Hz. For MBP, zero filling to 2048 points gave a digital resolution of 3.5 Hz; however, application of a cosine window function to the 750 complex points prior to Fourier transformation gave an effective resolution of 7243 Hz / 1500 points, or ~4.8 Hz.

NMR Data Extraction and Analysis

Data was analyzed using purpose-built software in Python and chemical shifts from the BMRB. Specifically, nmrPipe format spectra were directly read and 1D traces along the ^{13}C dimension of spin systems were extracted based on chemical shifts reported in the BMRB (Entry #7114) with small adjustments made due to minor chemical shift differences between our spectra and those reported.

Coupled to uncoupled peak height ratios were calculated after fitting of peaks to a "three-peak" model. Specifically, the Cα peak intensity at a point in the spectrum (x) was modeled as being composed of three Gaussian peaks; a central uncoupled peak (centered at m) assumed to be of greatest height (k_1), and two equally sized (height k_2) and spaced coupled peaks (distance d from the central peak) on either side of the central peak. Line width (s) was assumed to be equal for all peaks. Thus, an equation was derived for the three-peak model (1):

$$Intensity(x) = k_2 * e^{-\frac{(x-m-d)^2}{2s^2}} + k_1 * e^{-\frac{(x-m)^2}{2s^2}} + k_2 * e^{-\frac{(x-m+d)^2}{2s^2}} \quad (1)$$

Data over the width of a peak (~80 Hz, or 33 points for MBP HNCA spectrum, centered on the central, uncoupled peak) was considered for fitting. A curve fit function from Python was used to do the fitting. The parameters k_1 and k_2 were used to estimate the peak heights of the uncoupled, central peak and the coupled, adjacent peaks.

The assignment procedure was performed by first declaring an assignment as being unambiguous if, based on the known assignments, there was one and only one match between an internal chemical shift in a spin system and a sequential chemical shift in another spin system within a 'resolution' window. Resolution windows of 45 Hz (standard HNCA resolution) and 4.8 Hz (high resolution HNCA) were used. A check that the correct internal-sequential match had been made was also performed. Following assignment by chemical shift, all remaining ambiguous matches were evaluated for peak shape matching by calculating the correlation between an internal peak and all frequency matching sequential peaks. The highest correlation was declared the best match and then verified based on known assignments from the BMRB. Unambiguous assignments, and correct and incorrect assignments after pattern matching, were tallied. Systems that could not be sequentially assigned due to non-existent sequential systems (proline or exchange broadened systems) were counted separately.

Supplementary References:

1. Takeuchi, K.; Arthanari, H.; Shimada, I.; Wagner, G., Nitrogen detected TROSY at high field yields high resolution and sensitivity for protein NMR. *J Biomol NMR* **2015,** *63* (4), 323-31.

2. Richarz, R.; Sehr, P.; Wagner, G.; Wuthrich, K., Kinetics of the exchange of individual amide protons in the basic pancreatic trypsin inhibitor. *J Mol Biol* **1979,** *130* (1), 19-30.

3. Wagner, G., Characterization of the distribution of internal motions in the basic pancreatic trypsin inhibitor using a large number of internal NMR probes. *Quarterly Reviews of Biophysics* **1983,** *16* (1), 1-57.

4. Ottiger, M.; Bax, A., An Empirical Correlation between Amide Deuterium Isotope Effects on 13Cα Chemical Shifts and Protein Backbone Conformation. *Journal of the American Chemical Society* **1997,** *119* (34), 8070-8075.

5. Salzmann, M.; Pervushin, K.; Wider, G.; Senn, H.; Wuthrich, K., TROSY in triple-resonance experiments: new perspectives for sequential NMR assignment of large proteins. *Proc Natl Acad Sci U S A* **1998,** *95* (23), 13585-90.

6. Hyberts, S. G.; Milbradt, A. G.; Wagner, A. B.; Arthanari, H.; Wagner, G., Application of iterative soft thresholding for fast reconstruction of NMR data non-uniformly sampled with multidimensional Poisson Gap scheduling. *J Biomol NMR* **2012,** *52* (4), 315-27.

7. Delaglio, F.; Grzesiek, S.; Vuister, G. W.; Zhu, G.; Pfeifer, J.; Bax, A., NMRPipe: a multidimensional spectral processing system based on UNIX pipes. *J Biomol NMR* **1995,** *6* (3), 277-93.
